# Inhibition of neutral sphingomyelinase 2 impairs HIV-1 envelope formation and substantially delays or eliminates viral rebound

**DOI:** 10.1073/pnas.2219543120

**Published:** 2023-07-05

**Authors:** Seung-Wan Yoo, Abdul A. Waheed, Pragney Deme, Sehmus Tohumeken, Rana Rais, Matthew D. Smith, Catherine DeMarino, Peter A. Calabresi, Fatah Kashanchi, Eric O. Freed, Barbara S. Slusher, Norman J. Haughey

**Affiliations:** ^a^Department of Neurology, Johns Hopkins University School of Medicine, Baltimore, MD 21210; ^b^Virus-Cell Interaction Section, HIV-1 Dynamics and Replication Program, Center for Cancer Research, National Cancer Institute-Frederick, Frederick, MD 21702; ^c^Johns Hopkins Drug Discovery, Johns Hopkins University School of Medicine, Baltimore, MD 21205; ^d^Laboratory of Molecular Virology, George Mason University, Manassas, VA 20110; ^e^Psychiatry and Behavioral Sciences, Johns Hopkins University School of Medicine, Baltimore, MD 21210; ^f^Pharmacology and Molecular Sciences, Johns Hopkins University School of Medicine, Baltimore, MD 21210; ^g^Department of Oncology, Johns Hopkins University School of Medicine, Baltimore, MD 21224; ^h^Department of Neuroscience, Johns Hopkins University School of Medicine, Baltimore, MD 21205; ^i^Department of Medicine, Johns Hopkins University School of Medicine, Baltimore, MD 21210

**Keywords:** HIV-1, nSMase2, infection, viral loads

## Abstract

Although HIV-1 Gag is known to drive viral assembly and budding, the precise mechanisms by which the lipid composition of the plasma membrane is remodeled during assembly are incompletely understood. Here, we provide evidence that the sphingomyelin hydrolase neutral sphingomyelinase 2 (nSMase2) interacts with HIV-1 Gag and through the hydrolysis of sphingomyelin creates ceramide that is necessary for proper formation of the viral envelope and viral maturation. Inhibition or depletion of nSMase2 resulted in the production of noninfectious HIV-1 virions with incomplete Gag lattices lacking condensed conical cores. Inhibition of nSMase2 in HIV-1-infected humanized mouse models with a potent and selective inhibitor of nSMase2 termed PDDC [phenyl(R)-(1-(3-(3,4-dimethoxyphenyl)-2, 6-dimethylimidazo[1,2-b]pyridazin-8-yl) pyrrolidin-3-yl)-carbamate] produced a linear reduction in levels of HIV-1 in plasma. If undetectable plasma levels of HIV-1 were achieved with PDDC treatment, viral rebound did not occur for up to 4 wk when PDDC was discontinued. In vivo and tissue culture results suggest that PDDC selectively kills cells with actively replicating HIV-1. Collectively, this work demonstrates that nSMase2 is a critical regulator of HIV-1 replication and suggests that nSMase2 could be an important therapeutic target with the potential to kill HIV-1-infected cells.

## Significance

The mechanisms regulating remodeling of the plasma membrane required for HIV-1 assembly and budding are unknown. We found that the sphingomyelin hydrolase neutral sphingomyelinase-2 (nSMase2) interacts with HIV-1 Gag and regulates the hydrolysis of sphingomyelin to ceramide at plasma membrane HIV-1 assembly sites. Inhibition of nSMase2 results in the production of misshaped virions with increased sphingomyelin and reduced ceramide content that are not infectious. In HIV-1-infected humanized mice, inhibition of nSMase2 decreased plasma HIV-1, and if undetectable plasma HIV-1 was achieved, viral rebound did not occur. These results identify a host cell sphingomyelinase that is important for HIV-1 replication and is a druggable target for HIV-1.

HIV-1 assembly is driven by the viral Gag polyprotein precursor, several thousand molecules of which assemble at the inner leaflet of the host cell plasma membrane. Gag initially assembles to form a hexameric lattice that ultimately buds from the cell surface as an immature virus-like particle (VLP) of ~120 nm in diameter. During the assembly process, Gag recruits two single-stranded copies of the viral RNA genome and the GagPol polyprotein precursor, which contains the domains for the viral enzymes protease (PR), reverse transcriptase (RT), and integrase. Gag also recruits the viral envelope glycoproteins (Env) required for viral entry in the next round of replication. The matrix (MA) domain of Gag directs Gag to the plasma membrane where it anchors Gag in the lipid bilayer; MA also plays a key role in Env incorporation. The capsid (CA) domain drives Gag multimerization, the nucleocapsid (NC) domain binds viral genomic RNA, and the p6 domain recruits the cellular endosomal sorting complex required for transport machinery to promote the budding off of particles from the plasma membrane (for reviews, see refs. 1–3). The precise mechanism whereby the MA domain of Gag directs the trafficking of Gag to the plasma membrane to initiate VLP assembly is not completely understood but is known to involve the phospholipid phosphatidylinositol-4,5-bisphosphate (PIP_2_) that plays an important role in plasma membrane targeting of Gag through direct interactions with a highly basic patch of residues in MA (4–6). Gag multimerization at the plasma membrane is thought to trigger the exposure of a myristic acid moiety covalently attached to the N terminus of MA, leading to the insertion of the myristate into the inner leaflet of the lipid bilayer that anchors Gag in the membrane (5, 7, 8).

Concomitant with VLP release, the nascent particle undergoes maturation. Because PR functions as an obligate dimer, a requisite step in maturation is GagPol dimerization which leads to PR activation and proteolytic cleavage of Gag and GagPol. PR-mediated cleavage of Gag generates the mature Gag domains MA, CA, NC, and p6. The liberation of CA from the Gag polyprotein precursor allows CA to reassemble to form the viral core, which is composed of a conical outer shell of CA protein known as the CA. The CA is formed predominantly by a lattice of CA hexamers, with a total of 12 CA pentamers allowing the CA to form a closed structure. Viral genomic RNA and the viral enzymes RT and IN needed to carry out the early events in the next round of replication are packed inside the viral capsid (3, 9).

One of the least studied but key aspects of HIV-1 replication is remodeling of the plasma membrane at assembly sites that is required for viral assembly and envelope formation. Plasma membranes are not homogenous but instead are dynamic structures capable of creating and dismantling microdomains that have unique biophysical properties. Liquid-ordered (Lo) domains (sometimes called lipid rafts) are dynamic and contain a high content of sphingomyelin, saturated phospholipids, and cholesterol. The acyl chains of highly saturated lipids (such as sphingomyelin) are straight allowing for the lipids to pack closely together with limited lateral mobility in the membrane. These structures are stabilized by cholesterol that binds sphingomyelins together (*SI Appendix*, Fig. S1*A*). Liquid-disordered (Ld) regions of the plasma membrane are more fluid than Lo domains and contain a higher content of unsaturated lipids. The acyl chains of unsaturated lipids have multiple double bonds that create kinks in the acyl chains that prohibit close packing (*SI Appendix*, Fig. S1*B*). The HIV-1 envelope is enriched in cholesterol and several sphingolipids compared to the host cell plasma membrane, consistent with assembly and budding from Lo domains (10–12). Disrupting the structure of Lo domains by depleting cholesterol from cells severely impairs virus particle production (13, 14), presumably by reducing Gag binding and multimerization at the plasma membrane (15). There is an increasing amount of data suggesting that Gag does not simply target Lo domains but is an active participant in remodeling of the plasma membrane during viral assembly. In model membranes, Gag prefers to bind unsaturated 1,2-dioleoyl-*sn*-glycero-3-phosphocholine lipids compared with tightly packed saturated 1,2-distearoyl-*sn*-glycero-3-phosphocholine lipids (16). This reported preference of Gag for Ld regions of the membrane is not consistent with passive assembly in Lo domains. Some of the early modifications in the plasma membrane initiated by HIV-1 Gag are known. Gag has been reported to sense cholesterol and phospholipid acyl chain composition in a Gag–liposome binding assay (17) and to actively create PIP_2_- and cholesterol-enriched nanodomains at the inner leaflet of the plasma membrane during the early events of HIV-1 assembly (18–20). Using VLPs, it was recently proposed that this enrichment of PIP_2_ at the assembly site could influence the formation of nanodomains by interacting with outer leaflet lipids through the process of transbilayer coupling in which the C18 acyl chain of PIP_2_ interacts with saturated acyl chains of outer leaflet lipids such as sphingomyelin (21). Within these developing nanodomains, the intrinsic curvature of Gag polyproteins is thought to initiate budding through physical pressure on the membrane (22, 23). A continuous enrichment of proteins and saturated lipids during the early stages of assembly expands the Lo domain until further remodeling is not possible unless the membrane begins to curve (24). While the physical force of proteins can deform the plasma membrane to some extent, and the selective inclusion of proteins that prefer Lo domains contribute to membrane curvature and stability, the amount of curvature required for HIV-1 envelope formation requires a continuous remodeling of the lipid composition in plasma membranes during viral assembly (*SI Appendix*, Fig. S1 *D*–*G*). Precisely, how HIV-1 regulates plasma membrane remodeling to facilitate viral assembly and envelope formation is unknown.

One potential mechanism for rapid membrane remodeling during viral assembly is through a focal generation of ceramide. Lo domains rich in sphingomyelin can be converted to ceramide platforms (a gel-like phase; *SI Appendix*, Fig. S1*C*) by the actions of sphingomyelinases that remove the phosphorylcholine headgroup of sphingomyelin to create ceramide (25, 26). The biophysical properties of ceramide create a lateral phase separation in membranes, forming microdomains that sequester specific proteins referred to as ceramide-interacting proteins (21, 27). Ceramides are extremely hydrophobic and possess a marked intrinsic negative curvature that facilitates the formation of an inverted hexagonal phase and transmembrane lipid motion (flip/flop of lipids between the leaflets of a bilayer) (28–30). These properties are exploited by a number of viruses including influenza virus, hepatitis C virus, Ebola, and HIV-1 to regulate various aspects of viral replication including assembly and release (see ref. 31 for a review). For example, HIV-1 uses the exosome release pathway of cells for viral assembly and budding (32, 33). Based on evidence that exosome biogenesis is regulated by the formation of ceramide by the sphingomyelin hydrolase neutral sphingomyelinase 2 (nSMase2) (34, 35), we thought it possible that a similar mechanism is used by HIV-1 to remodel the plasma membrane. NSMase2 belongs to a family of hydrolases that are classified based on their pH optima as acidic, neutral, and alkaline (36). All known sphingomyelinases specifically hydrolyze the phosphocholine-headgroup of sphingomyelin to create ceramide.

Here, we demonstrate that nSMase2 plays a key role in HIV-1 Gag processing and particle maturation. Disruption of nSMase2 activity with the potent and selective small-molecule inhibitor PDDC (pIC50 = 6.57) (37), or by RNA interference, results in the production of HIV-1 virions with oddly shaped envelopes and an immature Gag lattice. This perturbation of HIV-1 biogenesis results in the dysfunction of lysosomes and ultimately the death of cells with actively replicating HIV-1. Inhibition of nSMase2 with PDDC in two different HIV-1-infected humanized mouse models produced a linear decrease of plasma viral loads and eliminated or substantially delayed viral rebound when inhibitor treatment was discontinued. This work together with findings reported in the companion paper (Waheed et al) identifies nSMase2 as an important cellular cofactor in the late stages of the HIV-1 replication cycle and as an antiretroviral (ARV) therapeutic target.

## Results

### NSMase2 Localizes with HIV-1 Gag in Lo Microdomains and Is Required for Proper Envelope Formation and Viral Maturation.

**Fig. 1.**
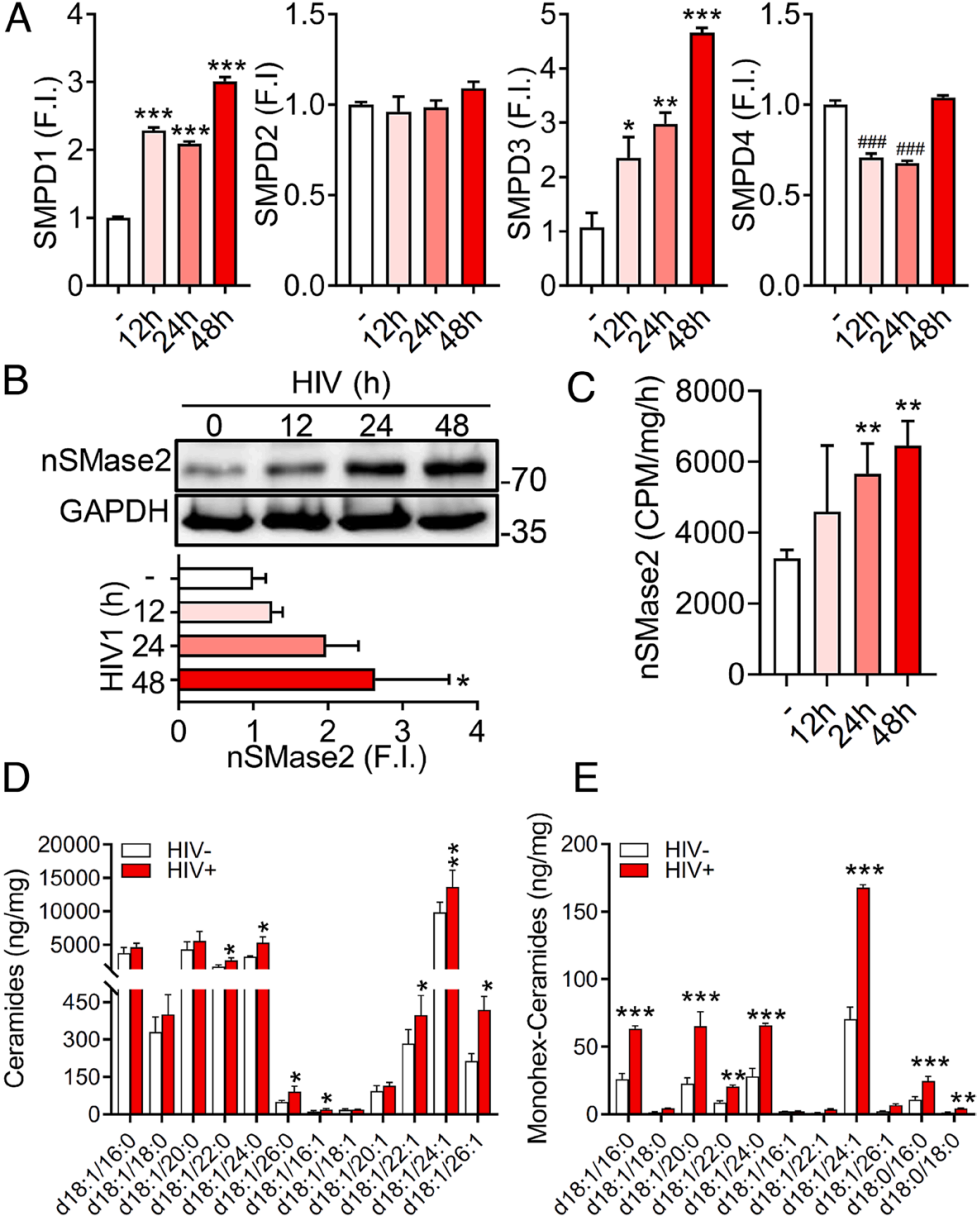
HIV-1 infection increases expression of nSMase2. (*A*) Transcriptional expression of the indicated sphingomyelinases following HIV_RF_ infection of H9 cells for 12 to 48 h relative to uninfected (white bar) cells (smpd1 = acidic; smpd2 = neutral-1; smpd3 = neutral-2; and smpd4 = neutral-3). FI denotes normalized fold induction of smpd genes compared to uninfected cultures. (*B*) Representative immunoblots (*B*, *Top*) and quantitation (*B*, *Bottom*) showing expression of nSMase2 at the indicated timepoints (0 to 48 h) following HIV_RF_ infection of H9 cells. (*C*) Quantitation of nSMase2 activity in H9 cells infected with HIV_RF_ for the indicated timepoints. (*D*) Quantitation of the indicated ceramides and (*E*) monohexosylceramides in HIV_RF_-infected H9 cells (48 h). Data are mean ± SD of n = 3 independent experiments/condition. *= *P* < 0.05, **= *P* < 0.01, and ***= *P* < 0.001 increased and ###= *P* < 0.001 reduced compared to uninfected controls. One-way ANOVA with Tukey’s post hoc analysis.

The sphingomyelin hydrolase nSMase2 plays an important role in regulating the size and stability of Lo membrane microdomains where the assembly of HIV-1 particles occurs (38). HIV-1 replication in H9 cells produced a time-dependent increase in the expression of smpd1 (acidic sphingomyelinase; aSMase) and smpd3 (nSMase2), but not smpd2 (nSMase1), or smpd4 (nSMase3) (Fig. 1*A*). The time-dependent increase in nSMase2 expression following HIV-1 infection of H9 cells was confirmed by western blot (Fig. 1*B*) and was accompanied by increased nSMase activity (Fig. 1*C*). Consistent with increased activity of nSMase2, several very-long-chain dihyroceramides and their corresponding ceramides including d18:1/22:1, d18:1/24:1, and d18:1/26:1 were increased during HIV-1 replicaiton (Fig. 1*D*). Likewise, the levels of several monohexosylceramides including d18:1/c16:0, d18:1/c20:0, d18:1/c24:0, and d18:1/c24:1 were increased during HIV-1 replication (Fig. 1*E*).

**Fig. 2.**
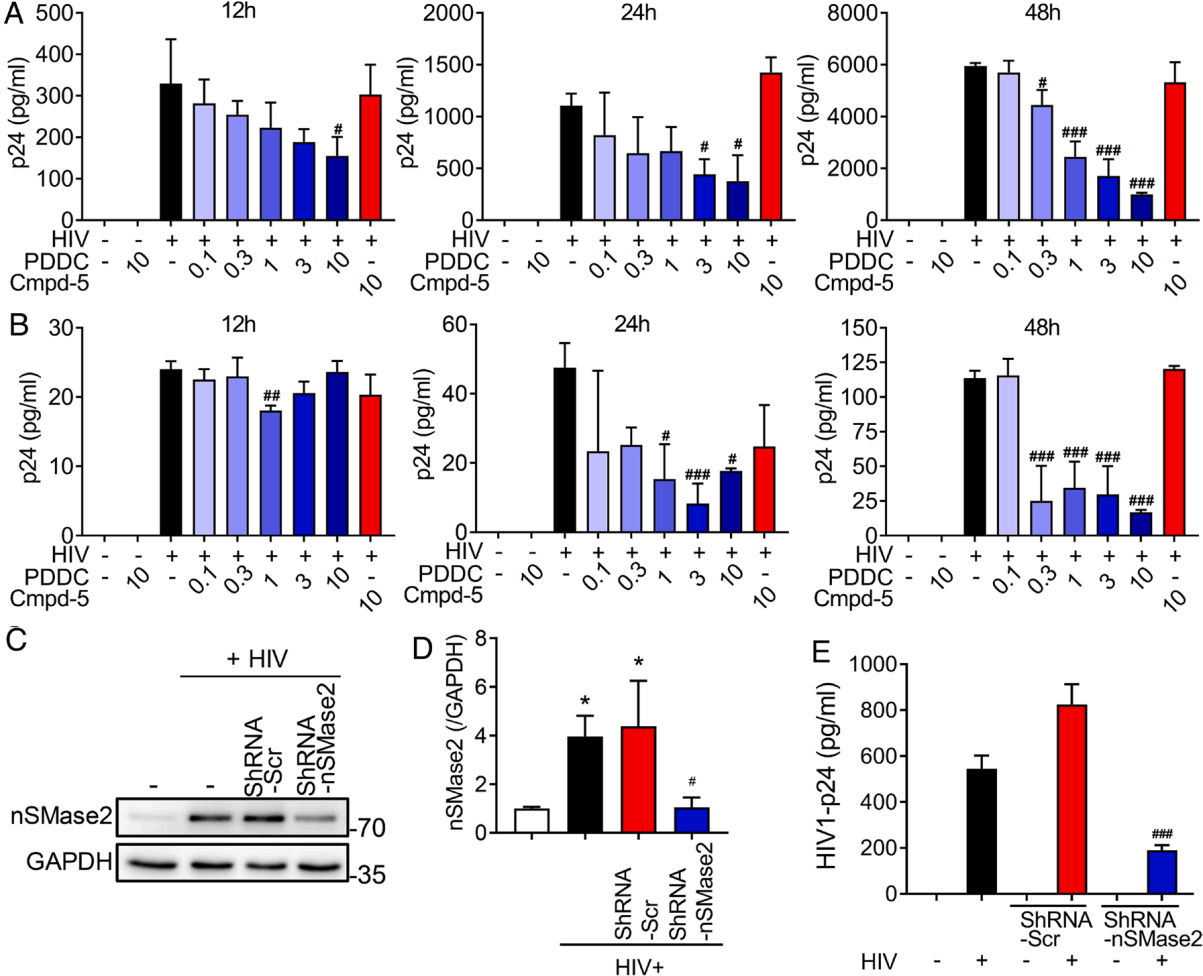
Pharmacologic inhibition or molecular interference of nSMase2 expression reduces extracellular levels of p24. H9 cells were infected with HIV_RF_, and primary human CD4+ cells were infected with HIV_MN_ for 7 d before experimentation. (*A*) Quantitation of p24 levels in media collected from H9 cells and (*B*) human primary CD4+ cells treated with a dose escalation of PDDC (0.1 to 10 µM) or an inactive structural analog of PDDC, Cmpd-5 (10 µM), and p24 levels in media were determined at the indicated timepoints (12 to 48 h). (*C*) Representative immunoblot and (*D*) quantitation showing molecular interference of nSMase2 protein expression in H9 cells. ShRNA targeting nSMase2 (shRNA-nSMase2) or scrambled shRNA (shRNA-Scr) were delivered to H9 cells in lentivirus vectors, and experiments were performed 48 h following HIV_RF_ infection. (*E*) Quantitation of p24 levels in media from HIV_RF-_infected H9 cells treated with shRNA-nSMase2 or shRNA-Scr. Data are mean ± SD of n = 3 independent experiments/condition. *= *P* < 0.05 compared to uninfected controls. #= *P* < 0.05, ##= *P* < 0.01, and ###= *P* < 0.001 compared to HIV-1 uninfected cells. One-way ANOVA with Tukey’s post hoc analysis.

Inhibition of nSMase2 with PDDC (37) produced a dose- and time-dependent reduction in extracellular levels of p24 in the HIV-1-infected T-cell line H9 (Fig. 2*A*) and primary T cells (Fig. 2*B*) as measured by p24 ELISA. A minimal effective concentration of 300 nM PDDC produced a 30% decrease in extracellular p24, and a 10 μM concentration produced an 83% reduction in extracellular p24 at the 48-h timepoint (Fig. 2 *A* and *B*). The structurally similar, but inactive, analog of PDDC, Cmpd5 (37), had no effect on p24 levels in HIV-1-infected H9 or primary T cells (Fig. 2 *A* and *B*). RNA interference of nSMase2 expression produced a similar reduction in levels of extracellular p24 in HIV-1-infected H9 cells (Fig. 2 *C*–*E*) confirming the direct involvement of nSMase2 rather than an off-target effect of PDDC. DPTIP, a structurally distinct inhibitor of nSMase2 (*SI Appendix*, Fig. S2*A*) (39), and the broadly used nSMase2 inhibitor GW4869 (*SI Appendix*, Fig. S2*B*) (40) also reduced extracellular levels of p24 in HIV-1-infected H9 cells, but the inactive des-hydroxyl analog of DPTIP (JHU3398) did not affect extracellular levels of p24 (*SI Appendix*, Fig. S2*A*). Inhibition of de novo ceramide synthesis with myrocin, which inhibits serine palmitoyl transferase (*SI Appendix*, Fig. S2*C*), or inhibition of the salvage pathway of ceramide production with fumonisin B1, which inhibits ceramide synthase (*SI Appendix*, Fig. S2*D*), did not reduce extracellular levels of p24.

**Fig. 3.**
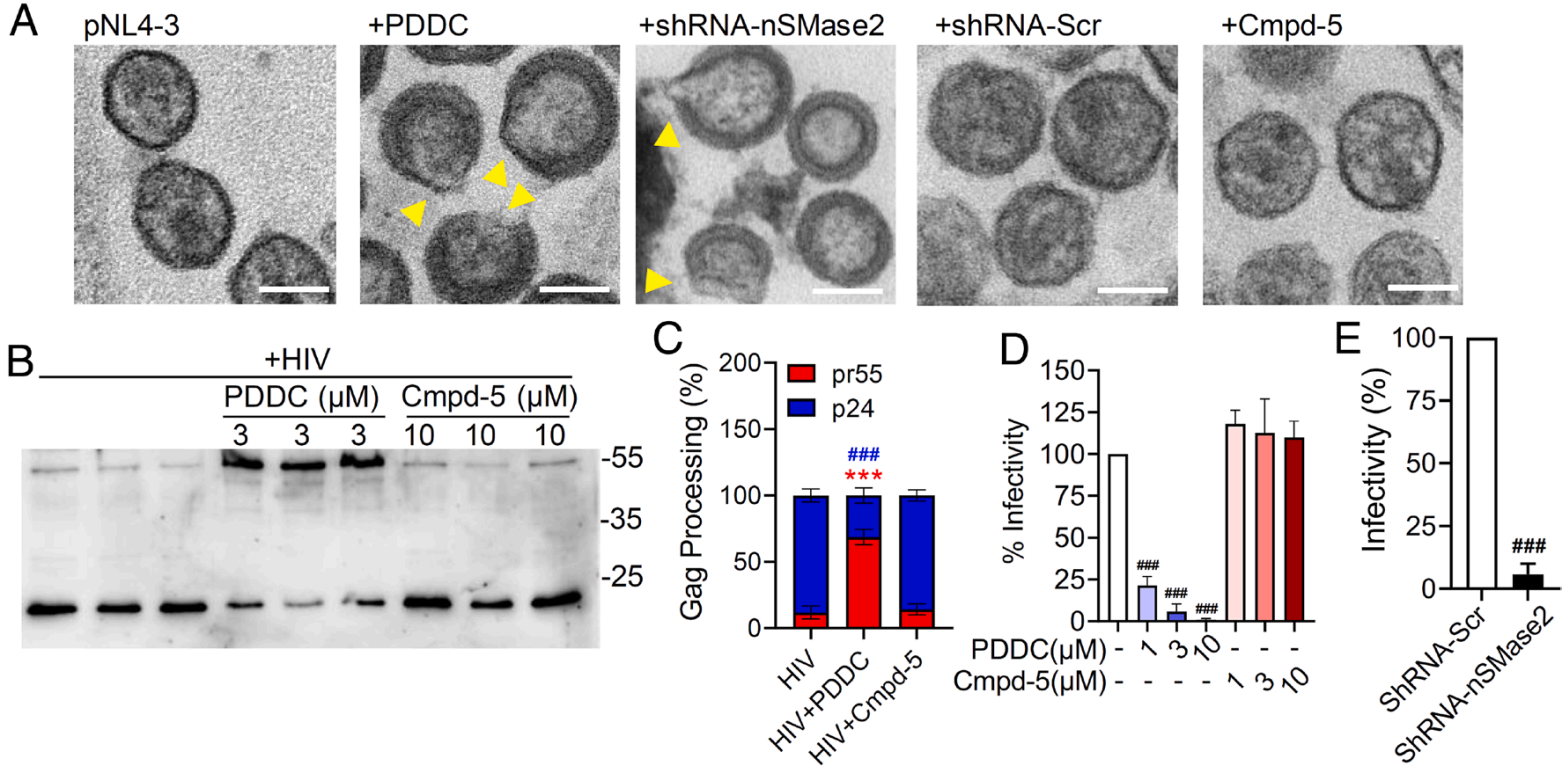
Pharmacologic inhibition or molecular interference of nSMase2 results in the release of HIV virions with irregularly shaped viral envelopes containing an immature Gag lattice. (*A*) Representative electron microscopy images of HIV-1 virions collected from culture media of HEK293T cells transfected with pNL4-3 and treated with PDDC (3 µM) or transduced with lentivirus expressing an shRNA targeting nSMase2 (shRNA-nSMase2) or a scrambled shRNA (shRNA-Scr). Data show that inhibition of nSMase2 by PDDC or molecular interference of nSMase2 expression results in deficits of HIV envelope formation (yellow triangles) and maturation of the Gag lattice. An inactive analog of PDDC, Cmpd-5 (10 µM), or shRNA-Scr did not modify the viral envelope or Gag maturation. (*B*) Representative western blot and (*C*) quantitation of Pr55Gag (red) and p24 (blue) in HIV-1 virions isolated from media of HIV_RF_-infected H9 cells treated with PDDC or Cmpd-5. (*D*) Quantitation of infectivity of HIV-1 virions collected from the media of HEK293T cells transfected with HIV_pNL4-3_ following inhibition of nSMase2 with PDDC or (*E*) pretreated with shRNA-nSMase2, demonstrating that blocking nSMase2 activity results in the production of noninfectious HIV-1 virions. Equal volumes of viral supernatants were used in the infectivity assays. Data are mean ± SD of n = 3 independent experiments/condition. ***(red) = *P* < 0.001 for pr55 HIV + PDDC compared to HIV, ### (blue) = *P* < 0.001 for p24 HIV + PDDC compared with HIV, and ### = *P* < 0.001 compared to untreated control or shRNA-Scr. One-way ANOVA with Tukey’s post hoc comparisons.

Despite our initial observation that pharmacological inhibition or knockdown of nSMase2 expression reduced extracellular p24, EM images of HIV-1 produced from 293T or HeLa cells transfected with the full-length HIV-1 molecular clone pNL4-3 showed that HIV-1 virions were still produced from cells treated with PDDC or following nSMase2 knockdown. However, these virions were oddly shaped and lacked condensed conical CA cores (Fig. 3*A*). Many of these particles contained large gaps in the immature Gag lattice and an irregular envelope with “tails” or “blebs” (Fig. 3*A*). Western blots confirmed that inhibition of nSMase2 with PDDC severely impaired the processing of Gag, while the inactive structural analog Cmpd-5 has no effect (Fig. 3 *B* and *C*). Similar results were obtained by knocking down the expression of nSMase2 using an shRNA directed against nSMase2 (*SI Appendix*, Fig. S3). These oddly shaped virions exhibited a severe reduction in infectivity (Fig. 3 *D* and *E*). These results demonstrate that the reduction of extracellular p24 following inhibition of nSMase2 is the result of a late, rather than an early block in the HIV-1 replication cycle that impairs proper envelope formation and Gag processing. The apparent discrepancy between the p24 ELISA assay, which showed reduced p24 release from cells in which nSMase2 was disrupted, and the western blot analysis, which showed a Gag processing defect but no reduction in particle production, was resolved by experiments showing that the p24 ELISA assay does not efficiently detect unprocessed Gag (*SI Appendix*, Fig. S3). Further details on deficits in Gag processing that occur when nSMase2 activity is disrupted can be found in the companion paper by Waheed et al.

Based on evidence that Lo membrane microdomains and ceramide are important determinants of HIV-1 particle binding and entry (41, 38), we next determined whether nSMase2 was required for HIV-1 entry into cells. TZM-bl cells containing a luciferase reporter gene under control of the HIV-1 long terminal repeat were treated with PDDC or the lentiviral-delivered shRNA targeting nSMase2 followed by infection with HIV-1_BAL_. We found similar levels of LTR activation in the presence and absence nSMase2 inhibition (*SI Appendix*, Fig. S2*E*). Following infection, inhibition of nSMase2 did not affect the expression of HIV-1 Env or TAR RNA (*SI Appendix*, Fig. S2 *F* and *G*). These data demonstrate that nSMase2 plays a role in HIV-1 replication but is not critical for HIV-1 entry, LTR activation, or gene transcription.

### NSMase2 Interacts with Gag and Is Present in HIV-1 Virions.

**Fig. 4.**
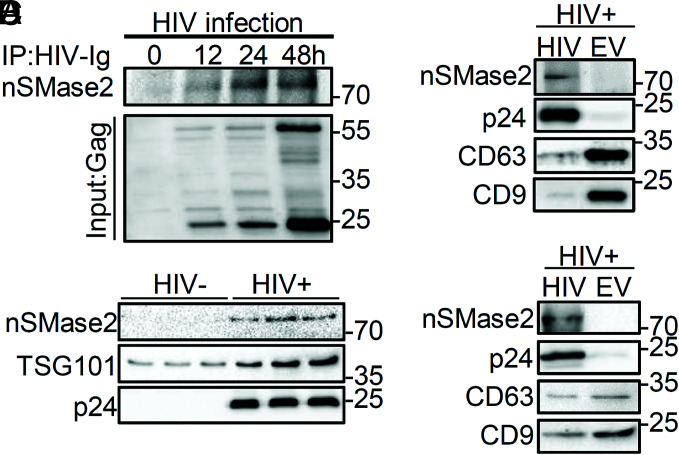
NSMase2 interacts with Gag and is packaged into HIV-1 virions. (*A*) Representative immunoblots showing interaction of nSMase2 with HIV-1 Gag. HIV_RF_-infected H9 cells were lysed; Gag was immunoprecipitated and subjected to SDS-PAGE, followed by immunoblotting with an antibody directed against nSMase2. Upper immunoblot demonstrates binding of Gag with nSMase2, and lower blot shows the amount of Gag input at each timepoint (0 to 48 h). (*B*) Representative immunoblots showing that particles isolated from the media of H9 cells infected with HIV_RF_ contained nSMase2, TSG101 and p24. Particles isolated from the media of uninfected H9 cells contained TSG101. (*C*) Media from HIV_RF_-infected H9 cells underwent ultracentrifugation, and HIV-1 was enriched by negative selection using magnetic beads coupled with antibodies to CD63 and CD9. This immunodepletion of EVs produced an HIV-1-enriched fraction that was immunopositive for nSMase2, p24, CD63, and CD9 and an EV-enriched fraction that was negative for p24 and nSMase2 but positive for CD63 and CD9. (*D*) Media from HIV_RF_-infected H9 cells underwent ultracentrifugation, and the HIV-1 fraction was enriched by positive selection using magnetic beads coupled with an antibody directed against gp120. The HIV-1-enriched fraction was nSMase2, p24, CD63, and CD9 positive, while the EV-enriched fraction was p24 and nSMase2 negative and CD63 and CD9 positive.

Based on published data that both Gag and nSMase2 target PIP_2_ (4–6, 42, 43), we next determined whether nSMase2 interacts with Gag. We found that nSMase2 coimmunoprecipitated with Gag (Fig. 4*A*), suggesting that these proteins physically interact. Using a multistep centrifugation procedure to enrich HIV-1 from supernatants, we found that nSMase2 and p24 were only detected in supernatants from HIV-1-infected H9 cells, whereas TSG101 was present in supernatants from both HIV-1-negative and HIV-1-infected cultures (Fig. 4*B*). The presence of TSG101 in supernatants of both HIV-1-negative and HIV-1-infected cultures suggests that EVs are coenriched with HIV-1 during ultracentrifugation. Since nSMase2 is known to regulate the production of at least one population of EVs (44), we confirmed this finding by binding CD63 and CD9 to magnetic beads to immunodeplete EVs from the supernatant of HIV-1-infected H9 cells and again observed that nSMase2 was present only in the HIV-1-enriched (unbound) fraction and not detected in the EV-enriched (bound) fraction (Fig. 4*C*). Although CD63 and CD9 are common markers for EVs, HIV-1 virions also contain these proteins (albeit at lower levels than EVs). To further validate that HIV-1 and not EVs carry nSMase2, we bound an antibody directed against gp120 to magnetic beads to immunodeplete HIV-1 from media of HIV-1-infected H9 cells. The HIV-1-enriched fraction (bound) contained nSMase2, p24, CD63, and CD9. The EV-enriched (unbound) fraction was negative for nSMase2 and p24 but positive for CD63 and CD9 (Fig. 4*D*). These data suggest that nSMase2 is predominantly packaged into HIV-1 virions and not EVs.

### nSMase2 Regulates the Sphingolipid Content of HIV-1 Virions.

**Fig. 5.**
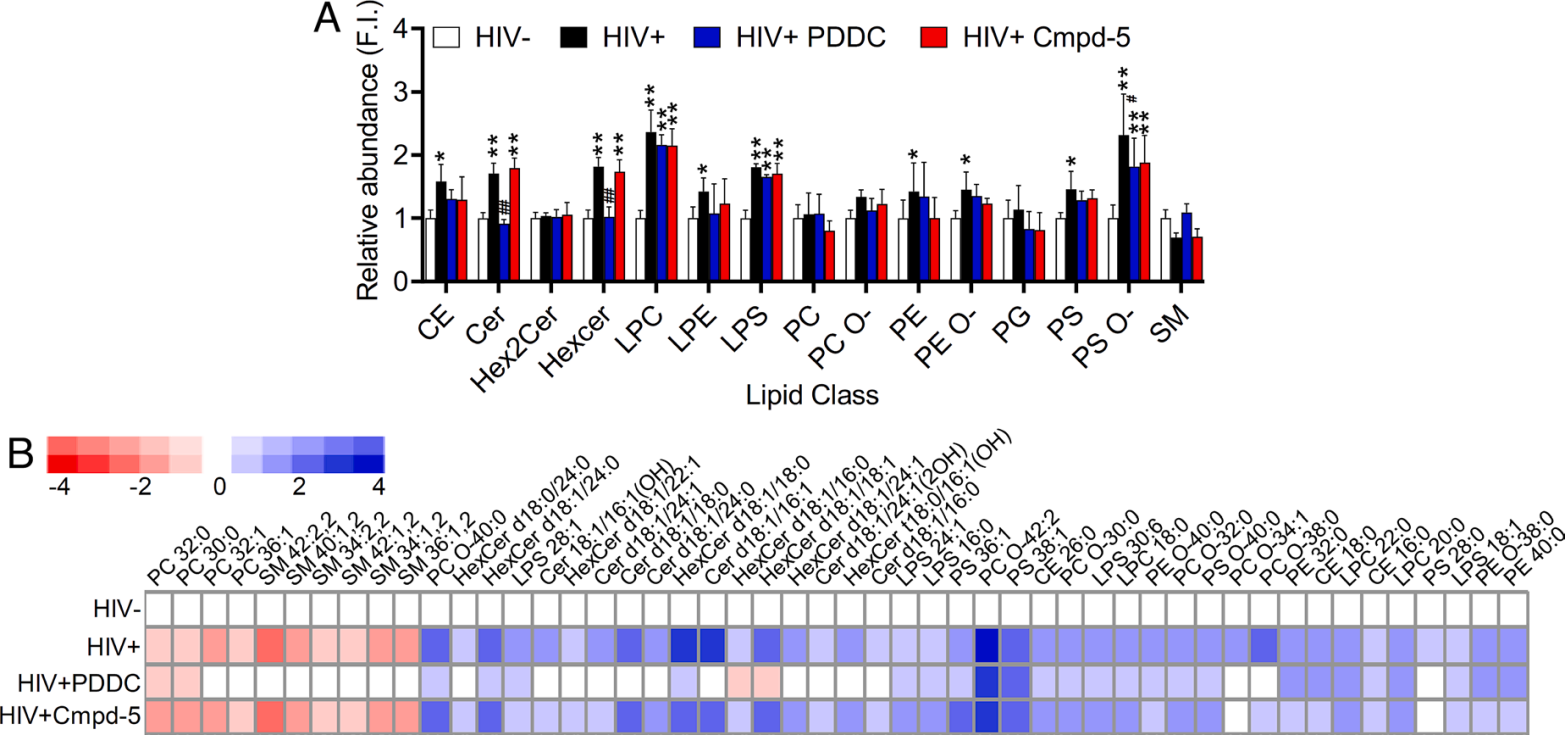
Inhibition of nSMase2 increases sphingomyelin and reduces the ceramide content of HIV-1 virions without modifying the content of cholesterol esters or phospholipids enriched in HIV-1 virions. (*A*) Quantitative analysis of the indicated lipid classes detected in HIV-1 virions collected from HIV_RF_-infected H9 cells treated with PDDC (3 µM) or the inactive structural analog Cmpd-5 (10 µM). Treatment of HIV_RF_-infected H9 cells with PDDC increased the content of sphingomyelins (SM) and reduced the content of ceramides and hexosylceramides in HIV-1 virions compared with EVs. Cmpd5 had no effect on the lipid content of HIV-1 virions. (*B*) Heat maps showing the amounts of individual lipid types in HIV-1 virions (HIV+), compared with particles collected from PDDC- and Cmpd5-treated cells relative to the lipid content of EVs isolated from uninfected cells (HIV−). Data are mean ± SD of n = 3 independent experiments/condition. *= *P* < 0.05 and **= *P* < 0.01 compared to EVs isolated from HIV- cells. # = *P* < 0.05 and ## = *P* < 0.01 compared to HIV+ cells. One-way ANOVA with Tukey’s post hoc comparisons.

Using the EV-enriched fraction as our baseline, we found that HIV-1 virions were enriched in cholesterol esters, ceramides, hexosylceramides, lysophosphatidylcholine, lysophosphati-dylethanolamine, lysophosphatidylserine, phosphatidylethanol-amine, oxidized phosphatidylethanolamine, phosphatidylserine, and oxidized phosphatidylserine with a strong trend toward decreased sphingomyelins (Fig. 5*A*). Inhibition of nSMase2 with PDDC reduced levels of cholesterol esters, ceramides, and hexosylceramides in HIV-1 virions to levels indistinguishable from EVs but did not alter levels of other phospholipids (Fig. 5*A*). The inactive structural analog of PDDC, Cmpd5, had no effect on any lipid classes enriched in HIV-1 (Fig. 5*A*). A more detailed analysis of the individual forms of lipids which either increased or decreased 1.5-fold or greater identified 50 individual lipids differentially expressed in HIV-1 particles compared to EVs. The HIV-1-enriched fraction contained lower amounts of long-chain sphingomyelins (n = 4 species) with increased amounts of ceramides (n = 7 species), hexosylceramides (n = 8 species), cholesterol esters (n = 3; CE; species), phosphatidyl serine (n = 2; PS; species), phosphatidyl ethanolamine (n = 2; PE; species), and lysophophatidyl serine (n = 3; LPS; species) (Fig. 5*B*). We also found that PCs (n = 3 species) and oxidized PC (n = 4; PC-O; species) were increased in HIV-1 virions compared with EVs (Fig. 5*B*). The only forms of PC we detected in virus particles were oxidized. Most of the phospholipids enriched in virions were saturated, consistent with the tight packaging required for a stable viral envelope. These data demonstrate that inhibition of nSMase2 modifies the ceramide and sphingomyelin content of HIV-1 particles and suggest that the abnormal properties of virions produced when nSMase2 activity is inhibited or expression is suppressed may be due to the reduced content of saturated sphingolipids and elevated content of lyso- and oxidized phospholipids that create a viral envelope with altered biophysical properties.

### Inhibition of nSMase2 Suppresses Plasma HIV-1 in Humanized Mice and Severely Delays or Eliminates Viral Rebound.

**Fig. 6.**
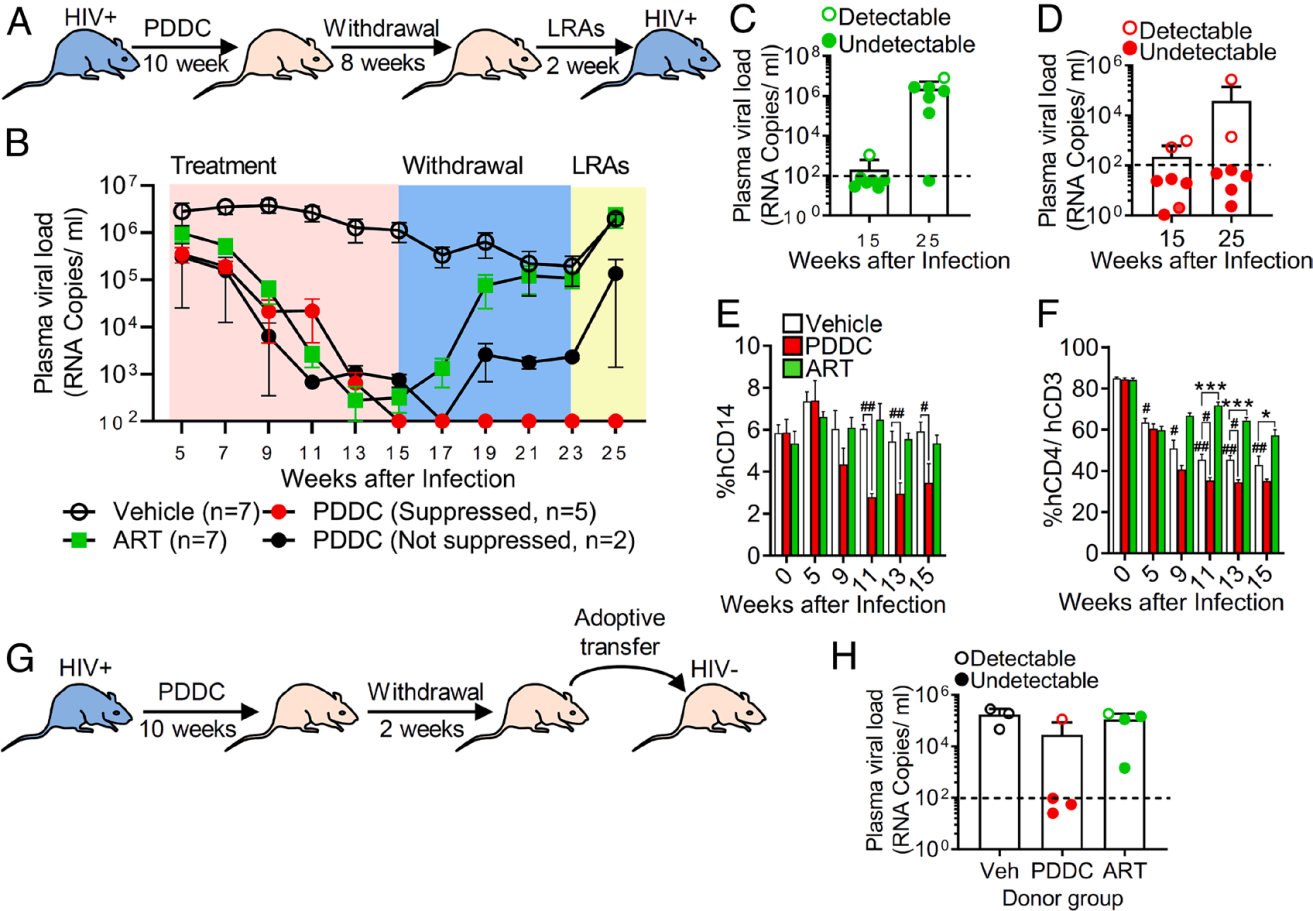
Inhibition of nSMase2 results in a linear decrease of plasma HIV-1 viral loads and prevents rebound in huNSG mice that achieved undetectable viral loads with PDDC treatment. (*A*) Experimental timeline for huNSG mice infected with HIV_ADA_ and treated with PDDC (I.P.,10 mg/kg, n = 7), antiretroviral therapy (ART; azidothymidine, lamivudine, and indinavir, IP, 45 mg/kg/d, n = 7), or vehicle (n = 7) for 10 wk followed by 8 wk of withdrawal and 2 wk of latency reversal (LRAs) with vorinostat (100 mg/kg, I.P.) and a bromodomain inhibitor (iBet, 20 mg/kg, I.P.). (*B*) Weekly plasma HIV viral loads for the indicated treatment conditions. Vehicle (open circle), ART (closed green square), PDDC with suppressed HIV-1 (red closed circles), and PDDC not fully suppressed HIV-1 (black closed circles). Plasma viral loads on the last day of treatment (day 15) and following LRAs (days 23 to 25) in (*C*) ART-treated mice and (*D*) PDDC-treated mice. Closed symbols denote mice that achieved plasma HIV viral loads below detection limits during PDDC treatment. Open symbols denote mice that did not achieve viral loads below detectable limits during the PDDC treatment period. (*E* and *F*) Quantitation of circulating human cells showing a reduction of *E* CD14+ and *F* CD4+/CD3+ cells in PDDC-treated mice compared to vehicle-treated mice. (*G*) Experimental timeline for adoptive transfer. Splenocytes were isolated from HIV_ADA_-infected huNSG mice 2 wk after treatment with ART or PDDC (drug withdrawal), and splenocytes were transferred to uninfected huNSG mice and recipient mice that were killed 6 wk after adoptive transfer (n = 3 to 4/group). (*H*) Plasma HIV-1 viral loads showing that splenocytes transferred from HIV-1-infected, ART-treated mice produced HIV-1 infection in recipient mice regardless of plasma viral loads of the donor mice. Splenocytes isolated from PDDC-treated mice with undetectable viral loads were unable to transfer infection compared with splenocytes isolated from HIV-infected PDDC-treated mice that had detectable plasma HIV viral loads. Closed symbols denote mice with viral loads below detection limits during PDDC treatment. Open symbols denote mice with detectable plasma viral loads during the PDDC treatment period. Data are mean ± SE. *= *P* < 0.05 and ***= *P* < 0.001 compared to vehicle. # = *P* < 0.05 and ## = *P* < 0.01 for the indicated comparisons.

To investigate the effect of inhibiting nSMase2 activity in HIV-1-infected humanized mouse models, we directly compared the ability of PDDC vs. an ARV regimen to suppress HIV-1 viral loads in HIV-1-infected NOD/SCID/IL2Rc-null (NSG) mice. Mice were administered PDDC at a dose previously shown to inhibit nSMase2 activity and block EV release in vivo (37), a combination of ARVs (azidothymidine, lamivudine, and indinavir), or vehicle for 10 wk. Inhibitors were then withdrawn for 8 wk followed by the administration of latency-reversing agents (LRAs; combination of vorinostat and iBet151) for an additional 2 wk (Fig. 6*A*). Vehicle-treated mice maintained a sustained plasma viral load for the duration of the experiment (Fig. 6*B* and *SI Appendix*, Fig. S4). PDDC treatment produced a linear decrease in plasma HIV-1 with five of seven mice below detection limits within 3 to 8 wk (Fig. 6*B* and *SI Appendix*, Fig. S4). The rate of reduction in plasma viral loads in mice administered ARVs was similar to that achieved with PDDC, with six of seven mice falling below detection limits (Fig. 6 *B* and *C* and *SI Appendix*, Fig. S4). PDDC did not alter body weight, autonomic nervous system function, somatosensory function, grip strength, or liver/kidney function (*SI Appendix*, Fig. S5 *A*–*L*). Following cessation of treatments, most of the mice administered ARVs exhibited rapid rebounds in plasma viremia (six of seven mice rebounded; Fig. 6*C*); in contrast, the majority of mice administered PDDC remained below detection limits (two of seven mice rebounded; Fig. 6*D* and *SI Appendix*, Fig. S4). In the PDDC treatment group, the two mice that exhibited viral rebound were those that did not achieve undetectable levels of plasma HIV-1 (Fig. 6*D* and *SI Appendix*, Fig. S4). LRAs increased the trajectory of viral rebound in mice with detectable viral loads regardless of treatment but had no effect on PDDC-treated mice that achieved undetectable viral loads (Fig. 6 *B*–*D* and *SI Appendix*, Fig. S4). There was a striking difference between the effects of ARVs compared with PDDC on immune cell numbers. With untreated HIV-1 infection, the number of CD14+ cells was stable, but the number of CD4+ cells declined with time of infection (Fig. 6 *E* and *F*). While ARVs preserved CD4+ cell numbers, PDDC reduced CD14+ and CD4+ cell numbers to below those observed with untreated HIV-1 infection (Fig. 6 *E* and *F*). Neither ARVs nor PDDC had an impact on the numbers of CD45+, CD3+, or CD19+ cells (*SI Appendix*, Fig. S6 *A*–*C*). To confirm that PDDC reduced the number of HIV-1-infected cells, we adoptively transferred splenocytes from HIV-1-infected mice to uninfected humanized mice (Fig. 6*G*). The adoptive transfer of splenocytes from vehicle- or ARV-treated mice to uninfected mice resulted in 100% transfer of HIV-1 infection (Fig. 6*H*). Infection was transferred from PDDC-treated mice with detectable plasma HIV-1 but not from those with undetectable viral loads (Fig. 6*H*). Together, these data demonstrate that inhibition of nSMase2 with PDDC reduces plasma HIV-1 loads, and if undetectable plasma levels are achieved, viral rebound is not apparent for at least 2 wk following inhibitor withdrawal. The selective decline in CD4+ and CD14+ cells observed with PDDC treatment suggests that inhibition of nSMase2 may eliminate HIV-1-infected cells.

**Fig. 7.**
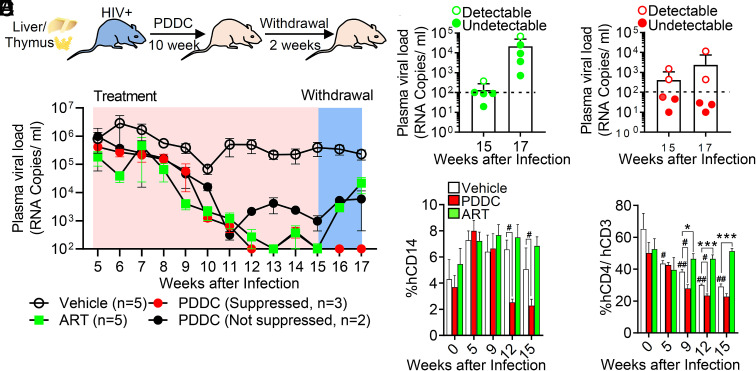
Inhibition of nSMase2 reduces HIV-1 viral loads and prevents rebound in BLT mice that achieved undetectable viral loads with PDDC treatment. (*A*) Experimental timeline for BLT mice infected with HIV_ADA_ and treated with chow containing PDDC (500 mg/Kg) or chow containing a combination of antiretrovirals (tenofovir disoproxil fumarate, 1,560 mg/kg; emtricitabine, 1,500 mg/kg; and raltegravir, 600 mg/kg) for 10 wk followed by withdrawal for 2 wk. (*B*) Weekly plasma viral loads for the indicated treatment conditions in HIV-infected BLT mice. Vehicle (open circle), ART (closed green square), PDDC with suppressed HIV-1 (red closed circles), and PDDC not fully suppressed HIV-1 (black closed circles). Plasma viral loads on the last day of treatment (day 15) and following drug withdrawal (day 17) in (*C*) ART-treated mice and *D* PDDC-treated mice. Closed symbols denote mice that achieved plasma viral loads below detection limits during inhibitor treatment. Open symbols denote mice that did not achieve viral loads below detectable limits during the treatment period. (*E* and *F*) Quantitation of human CD14+ cells and CD4+/CD3+ cells for the indicated treatment conditions and timepoints showing a time-dependent reduction of *E* CD14+ and *F* CD4+/CD3+ cells in HIV-infected mice treated with PDDC. Data are mean ± SE. *= *P* < 0.05 and ***= *P* < 0.001 compared to vehicle. # = *P* < 0.05 and ## = *P* < 0.01 for the indicated comparisons.

To examine the ability of PDDC to suppress HIV-1 replication in a second humanized mouse model, we evaluated the effect of nSMase2 inhibition in HIV-1-infected bone marrow–liver–thymus (BLT) mice. Infected mice were fed chow containing PDDC (50 mg/kg) or ARVs (combination of TDF, FTC, RAL) for 10 wk followed by 2 wk of drug withdrawal (Fig. 7*A*). The dose of PDDC-containing chow chosen was previously shown to inhibit nSMase2 and block EV release in vivo (45). As observed in the HIV-1-infected NSG mice, PDDC produced a linear decrease in plasma HIV-1 with a trajectory of decline similar to ARV treatment (Fig. 7*B* and *SI Appendix*, Fig. S7). Plasma HIV-1 fell to below detectable limits in 3 of 5 mice in the PDDC treatment group and four of five mice in the ARV group (Fig. 7 *B*–*D* and *SI Appendix*, Fig. S7). All mice in the ARV treatment group exhibited a rapid viral rebound (Fig. 7 *B* and *C* and *SI Appendix*, Fig. S7). None of the mice in the PDDC group that achieved plasma viral loads below detectable limits exhibited viral rebound following inhibitor withdrawal (Fig. 7 *B* and *D* and *SI Appendix*, Fig. S7). With untreated HIV-1 infection, the numbers of CD14+ cells in HIV-1-infected BLT mice were relatively stable, while the number of CD4+ cells declined with duration of replication (Fig. 7 *E* and *F*) consistent with replication-induced death of CD4+ cells. ARV treatment prevented the decline in CD4+ cell numbers in HIV-1-infected BLT mice (Fig. 7 *E* and *F*). In HIV-1-infected BLT mice fed PDDC-containing chow, CD14+ and CD4+ cell numbers were reduced below the levels observed with untreated HIV-1 infection (Fig. 7 *E* and *F*). ARVs or PDDC did not reduce the numbers of CD45+, CD3+, or CD19+ cells (*SI Appendix*, Fig. S8 *A*–*C*). These data confirm in a second model of HIV-1 replication that inhibition of nSMase2 with PDDC can reduce plasma HIV-1 loads to below detectable limits. The selective declines in CD4+ and CD14+ cells observed with PDDC treatment suggest that inhibition of nSMase2 may eliminate HIV-1-infected cells.

### Inhibition of nSMase2 Selectively Promotes Cell Death in Infected Cells with Actively Replicating HIV-1.

**Fig. 8.**
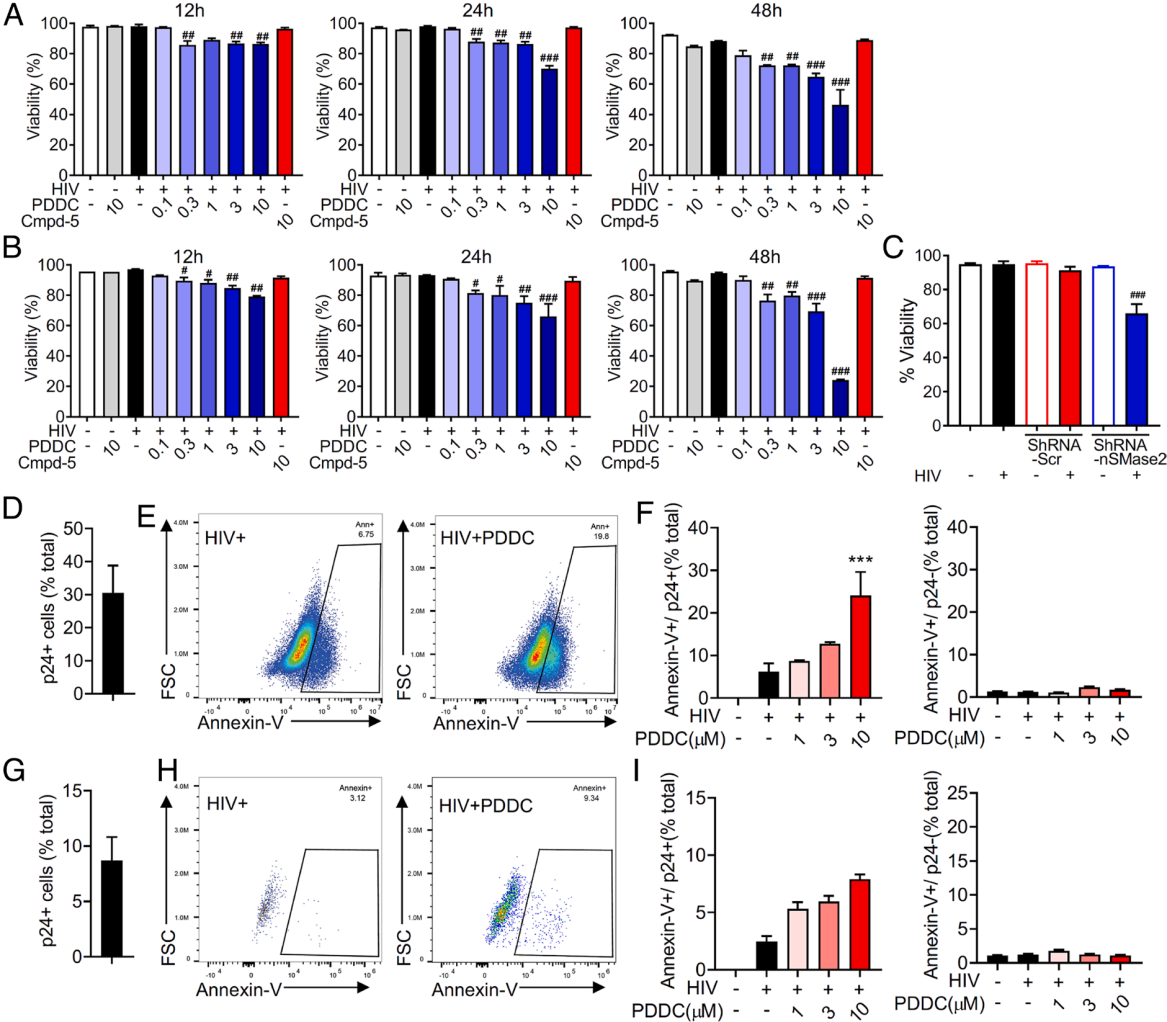
Inhibition of nSMase2 selectively kills HIV-1-infected cells. (*A*) Quantitation of cell viability in H9 cells and (*B*) human primary CD4+ cells. H9 cells were infected with HIV_RF_and primary human CD4+ cells were infected with HIV_MN for 7 d and then_ treated with a dose escalation of PDDC (0.1 to 10 µM) or the inactive structural analog of PDDC, Cmpd-5 (10 µM), and cell viability was determined at 12 to 48 h following treatment with PDDC. (*C*) Quantitation of cell viability following knock down of nSMase2 expression with a vector expressing an shRNA targeting nSMase2 (shRNA-nSMase2) or a scrambled shRNA (shRNA-Scr) 48 h following infection of H9 cells with HIV_RF._ (*D*) Percent of HIV-1-infected H9 cells. (*E*) Representative flow cytometry plots and (F) quantitation of annexin V+ and p24+ dual immunopositive H9 cells. (*G*) Representative flow cytometry plots for annexin V+ and p24- cells. (*H*) Quantitation of annexin V+ and p24- H9 cells treated with PDDC (0 to 10 µM). (*I*) Percent of HIV-1-infected primary CD4+ cells. Data are mean ± SD of n = 3 independent experiments per condition. ***= *P* < 0.001 compared to the untreated HIV-1-infected group. # = *P* < 0.05, ## = *P* < 0.01, and ### = *P* < 0.001 compared to the untreated HIV-infected group. One-way ANOVA with Tukey’s post hoc comparisons.

We next determined whether cell viability was affected by inhibition of nSMase2 with PDDC. We observed a dose- and time-dependent decrease in the viability of HIV-1-infected H9 and primary T cells treated with PDDC (Fig. 8 *A* and *B*). However, the highest dose of PDDC tested had no impact on the viability of uninfected H9 or primary T cells. The inactive structural analog of PDDC, Cmpd5, had no impact on the viability of HIV-1-infected or uninfected H9 or primary T cells (Fig. 8 *A* and *B*). NSMase2 knockdown also reduced the viability of HIV-1-infected H9 cells but did not reduce the viability of uninfected cells (Fig. 8*C*). We next determined that PDDC treatment of HIV-1-infected H9 cells (Fig. 8*D*) produced a dose-dependent increase in the percent of annexin V+/p24+ cells (Fig. 8 *E* and *F*) but had no impact on the percent of annexin V+/p24-negative cells (Fig. 8 *G* and *H*). A similar result was obtained with FACS analysis of HIV-1-infected primary T cells (Fig. 8 *H*–*M*). In constitutively HIV-1-infected U1 cells that exhibit a low baseline level of HIV replication, PDDC did not reduce cell viability at any of the concentrations tested. Induction of HIV replication in U1 cells by TNFα dramatically increased the amount of HIV produced, and these cells became susceptible to death induced by PDDC (*SI Appendix*, Fig. S9). Together, these data suggest that inhibition of nSMase2 promotes an apoptotic phenotype in HIV-1-infected cells with actively replicating HIV-1.

## Discussion

While we understand a great deal about HIV-1 assembly, budding, and maturation, we know relatively little about how the viral envelope is formed. Our data support the concept that HIV-1 actively modifies the lipid composition of the plasma membrane by complexing Gag with nSMase2 to generate ceramide at HIV-1 assembly sites. Several studies have examined the lipid composition of HIV-1 compared to the host cell of origin and found that the lipid composition of HIV-1 is distinct from the overall lipid composition of the cell of origin and more closely resembles the Lo regions of the host cell membrane (10, 46–48). Ceramide appears to be enriched in HIV-1 virions but is a relatively minor component of the viral envelope compared to other types of lipids (46). Nevertheless, our data demonstrate that the focal generation of ceramide by nSMase2 is critical for proper formation of the HIV-1 envelope and for viral maturation.

Shortly after its synthesis in the cytosol, HIV-1 Gag targets the plasma membrane by binding to PIP_2_ and potentially other negatively charged lipids on the inner leaflet of the plasma membrane and via the insertion of a covalently linked myristic acid into the lipid bilayer. This increases the affinity of Gag for the membrane by ~100-fold (49). A direct interaction between basic residues in the MA domain of HIV-1 Gag and PIP_2_, and potentially other phosphatidylserine-containing lipids, stabilizes the interaction of Gag with the inner leaflet of the plasma membrane (4, 5, 13). The self-assembly of Gag has been suggested to be the driving force in PIP_2_/cholesterol-enriched lipid nanodomain formation with cholesterol facilitating myristate insertion and PIP_2_ binding (20, 49, 50). The CA domain regulates Gag self-assembly into hexamers that form a spherical immature Gag lattice. This intrinsic curvature of proteins is thought to initiate budding through physical pressure on the membrane (22, 23). Subsequently, Gag is proposed to facilitate the formation of PIP_2_/cholesterol nanoclusters in the plasma membrane (50). Cholesterol interacts with sphingomyelin in Lo microdomains (51), suggesting that the anchoring of Gag complexed with nSMase2 to the inner leaflet of the plasma membrane would initiate sphingomyelin hydrolysis and the ceramide produced would displace cholesterol during or following Gag self-assembly. Biophysical studies using artificial membrane preparations have demonstrated that ceramides displace sterols from Lo membrane microdomains (52, 53) and nSMase2 activity has been shown to promote the transport of cholesterol to the endoplasmic reticulum for esterification (54). This generation of ceramide within Lo domains alters the biophysical properties of the membrane and results in the formation of large, ceramide-enriched platforms with a gel-like structure**.** It has been demonstrated using artificial membranes containing low amounts of cholesterol that ceramide segregates together with sphingomyelin into small ceramide/sphingomyelin-gel domains. With increasing amounts of cholesterol, the ceramide/sphingomyelin gel domains are reduced, the membrane becomes more rigid, and a Lo phase predominates (53, 55). This is consistent with previous reports that found the HIV-1 membrane to be tightly packed but less ordered than the rigid Lo domains isolated from host cells (46). While the function of this focal generation of ceramide during viral assembly remains to be defined, it is likely that a slightly less rigid structure is required for the plasma membrane to wrap the budding virus, a notion that is supported by the known properties of ceramide to form negative curvatures by promoting a lamellar–hexagonal phase transition (56, 57). Together, these data suggest that HIV-1 Gag targets Lo membrane microdomains at the inner surface of the plasma membrane where nSMase2 hydrolyzes sphingomyelin to ceramide. This focal modification in the lipid composition of the membrane alters the biophysical properties of Lo domains to create gel-like domains that we speculate are required for activation of the viral PR. Precisely, when and by what mechanism nSMase2 associates with Gag are actively being investigated.

NSMase2 belongs to a family of hydrolases that are classified based on their pH optima as acidic, neutral, and alkaline (36). All known sphingomyelinases specifically hydrolyze the phosphocholine headgroup of sphingomyelin to form ceramide. NSMase2 contains an N-terminal domain, a cytoplasmic juxtamembrane region, an insertion region, and a catalytic domain. The N-terminal domain is an integral membrane region containing two hydrophobic segments that serve to anchor nSMase2 to the membrane (58) and an allosteric activation domain that binds phosphatidylserine (59–61). Binding of phosphatidylserine to the N-terminal domain results in a conformational shift mediated through the juxtamembrane regions that facilitates an interaction of the N-terminal domain with the catalytic domain at the interface of the plasma membrane. This conformational shift removes what is known as the DK loop from the enzyme’s active site, allowing sphingomyelin into the active site for hydrolysis (62). Exactly what promotes the conformational shift required for nSMase2 activation is not fully understood but appears to involve serine phosphorylation and protein–protein interactions. The mammalian form of nSMase2 contains a binding motif for the phosphatase calcineurin that is located on the periphery of the catalytic domain (63). Phosphorylation by calcineurin enhances nSMase2 activity as demonstrated by mutation of the calcineurin binding site that results in constitutive phosphorylation and increased activity of nSMase2 (63). Activity by protein–protein interaction has been demonstrated for the inflammatory cytokine TNF-α that activates nSMase2 by recruiting it to the plasma membrane through interactions between the catalytic domain of nSMase2 and a multiprotein complex of the TNF receptor–FAN–RACK1–EED (64–66). It is not clear at this time whether the interaction between nSMase2 and Gag is direct or involves a protein complex. Based on what is currently known about the mechanisms for nSMase2 activation, it is likely that the interaction with Gag (either direct or indirect) is sufficient to induce the conformational shift and possibly phosphorylation required for nSMase2 activation.

A striking finding in this study is the impact of nSMase2 inhibition on HIV-1 replication and the survival of HIV-1-infected cells. As expected, HIV-1-infected humanized mice treated with ART exhibited viral rebound when ART was withdrawn and rebound occurred regardless of plasma viral load status at the time of ART withdrawal. In contrast, none of the mice treated with PDDC that achieved plasma viral loads below detectable limits exhibited viral rebound when PDDC was withdrawn, even following the administration of LRAs. This lack of viral rebound was accompanied by a selective reduction of CD14+ and CD4+ cells, suggesting that PDDC may have selectively eliminated HIV-1-infected cells. This conclusion is further supported by evidence that HIV-1-infected cells exposed to PDDC expressed the apoptotic marker phosphatidylserine on their surface to a far greater extent than untreated HIV-1-infected cells. The mechanism(s) for this severe delay or absence of viral rebound is actively being investigated in our laboratories.

In this proof-of-concept study, we identify the sphingomyelin hydrolase nSMase2 as a critical regulator of HIV-1 biogenesis and maturation. Inhibition of this enzyme appears to promote the death of HIV-1-infected cells. These observations raise a number of additional questions to address in future studies. When and precisely how does HIV-1 Gag interact with nSMase2 and is this interaction sufficient to induce enzyme activity? Is there a function for nSMase2 in HIV-1 virions and why is nSMase2 required for HIV-1 maturation? Can HIV-1 escape inhibition of nSMase2? Precisely how does inhibition of nSMase2 kill HIV-1-infected cells and can latently infected cells be targeted for elimination? These and other questions are actively being pursued by our laboratories.

## Materials and Methods

### Cell Culture and Treatments.

The human H9 T-cell line was infected with 100 ng/mL of HIV-1-RF at 37 °C for 4 h and plated at a density of 2.0 × 10^5^ /mL for experimentation. Primary human CD4+ cells were isolated as previously reported (67). Deidentified blood from healthy donors was diluted 1:5 in phosphate buffered saline (PBS) containing 5 mM ethylenediaminetetraacetic acid (EDTA) and 2% FBS, layered gently over Ficoll (GE Healthcare Biosciences, Marlborough, MA) in SepMate TM 50-mL tubes (Stem Cell Technology Vancouver, BC, Canada), followed by centrifugation at 1,200 *g* for 10 min at room temperature. The PBMC fraction was centrifuged at 300 *g* for 8 min and incubated in red blood cell lysis buffer (155 mM NH_4_Cl, 10 mM KHCO_3_, and 1 mM EDTA pH 7.2) at 37 °C for 10 min. CD4+ T cells were isolated from PBMCs using the EasySepTM Human Naïve CD4+ T Cell Isolation Kit (Stem Cell Technology). Primary CD4+ cells were infected with HIV-1-MN (200 ng/mL, NIH/AIDS repository) and incubated overnight at 37 °C. HIV-1 infection of CD4+ and H9 cells was allowed to develop for 7 d prior to experimentation. H9 cells were treated with a dose range of the nSMase2 inhibitor PDDC (0.1, 0.3, 1, 3, and 10 µM), the inactive structural analog compound 5 (Cmpd-5, 10 µM), serine palmitoyl transferase inhibitor myriocin (0.1, 0.3, 1, 3, and 10 µM, Sigma), ceramide synthase inhibitor fumonisin B1 (0.1, 0.3, 1, 3, and 10 µM, Tocris, Minneapolis, MN), zVAD-FMK (50 µM, Tocris), or vehicle. Primary CD4+ cells were treated with the same dose range of PDDC, Cmpd-5, or vehicle for 30 min prior to stimulation with phytohemagglutinin (PHA, 1 μg/mL, Sigma)/IL-2 (100 IU/mL, Sigma) in primary CD4+ cells. HIV-1 p24 was measured by ELISA (Perkin Elmer, Akron, OH) 12, 24, and 48 h after treatment using the manufacture’s protocol. Culture media (200 µL) were collected at each timepoint and centrifuged at 500 *g* for 5 min to clear any cells or debris. Cell culture media were placed into a p24 antibody–coated plate and incubated at 37 °C for 2 h, washed with the manufacturer’s washing buffer, and then incubated with the detection antibody (100 µL) at 37 °C for 1 h. Streptavidin–HRP was added for 30 min, and the reaction was visualized using the chromogenic substrate, ortho-phenylenediamine, at 420 nm OD on a SpectraMax M2 microplate reader (Molecular Devices, Sunnyvale, CA). Cell viability was measured by trypan blue staining at 12, 24, and 48 h after treatments. HIV-1 transcriptional activity was measured in TZM-bl cells (NIH AIDS Reagent Program) containing a luciferase gene under control of the HIV-1 LTR (ref). TZM-bl cells were plated at a density of 5.0 × 10^4^/mL in 96-well plates and infected with 20 ng/mL of HIV-1-Bal. Luciferase activity was measured using the Luciferase Assay System (Promega, WI, USA) according to the manufacturer’s protocol and read on a Fluoroskan Ascent FL luminometer (Thermo Fisher Scientific). H9 cells or primary CD4+ cells were treated with PDDC (1, 3, and 10 µM) for 24 h following HIV-1 infection as described above. The cells were stained with annexin V (BioLegend, San Diego, CA), fixed with 4% paraformaldehyde, and permeabilized with membrane permeabilization buffer (BioLegend). Cells were stained with a customized HIV-1-Gag antibody (NIH AIDS Reagent Program) conjugated with Alexa 532 using an antibody labeling kit (Thermo Fisher Scientific) and analyzed by flow cytometry, Cytek Aurora (Fremont, CA)

### Genetic Knock Down of nSMase2.

Genetic knock down of nSMase2 expression in HIV-1-infected cells was accomplished by transduction of lentivirus expressing shRNA targeting nSMase2. H9 cells were transduced with an MOI of five piLenti-sh-nSMase2 in 1 µg/mL of polybrene containing four different shRNAs targeting nSMase2: 5′-GCCCTTATCTTTCCATGCTA CTGGCTGGT-3′; 5′-GCCACCAAATTGAAAGAGCAGCTGCACGG-3′; 5′-CCAAAGAATCGTCG GGTACATCGCCTGCA-3′; and 5′-ACACTCCCTGTTCACCCACTACAGGGACC-3′. Genetic knockdown of acidic sphingomyelinase (aSMase) was accomplished by lentivirus expressing four different targeting shRNA for aSMase: 5′-CTGTGCAATCTGCTGAAGATAGCACCACC-3′; 5′-CCGCCTCATCTCTCTCAATATGAATTTTT-3′; 5′-GGCCACACTCATGTGGATGAATTTGAGGT-3′; and 5′-CCAGACCTTCTGGTTTCTCTACCATAAGG-3′. Lentivirus expressing scrambled shRNA (piLenti-scrambled RNA) was used as a control. Twenty-four h after transduction of cells with lentivirus, cells were collected and infected with HIV-1-RF (100 ng/mL, H9 cells), or stimulated with TNF-α (500 ng/mL, U1 cells). Forty-eight h later p24 levels were measured as described above.

### Pharmacological Inhibition of nSmase2.

PDDC and compound five were synthesized in-house using methods we previously described (CITE BJP 2019 paper). Both compounds were characterized by 1H/13C NMR for structural identification and confirmed to be of ≥95% purity by LC/MS.

### Immunoblotting.

HIV-1-infected cells were lysed with RIPA buffer containing 50 mM Tris–HCl (pH 7.5), 150 mM NaCl, 10 mM EDTA, 2 mM EGTA, 50 mM NaF, 0.5% sodium dodecyl sulfate (SDS), and 1% NP-40 supplemented with PR inhibitor cocktails (Roche Diagnostics Corporation, Indianapolis, IN). Soluble proteins were resolved by 10% sodium dodecyl sulfate-polyacrylamide gel electrophoresis (SDS-PAGE) and transferred to poly(vinylidene fluoride) (PVDF) membranes (BioRad, Hercules, CA, USA) as previously reported (68). Nonspecific binding sites were blocked with 5% (w/v) milk in TBS containing 0.1% Tween 20 (TBS-T, v/v). After blocking, blots were incubated overnight at 4 °C with a primary antibody for nSMase2 (1:1,000, ECM Biosciences), HIV-11-p24 (1:1,000, NIH AIDS Reagents), TSG101 (1:1,000, Cell Signaling), CD63 (1:1000, Santacruz, Dallas, TX), β-actin (1:5000, Sigma), or GAPDH (1:5000, Sigma). Following three washes with TBS-T, blots were incubated for 1 h at room temperature with the appropriate IgG HRP-linked antibody (1:2000; Cell Signaling Technology, Danvers, MA) and developed by enhanced chemiluminescence (Sigma). Image acquisition and analysis were performed using a G:BOX Imaging System (Syngene, Frederick, MD).

### Electron Microscopy.

Cells were fixed with 4% paraformaldehyde (PFA), 2% glutaraldehyde, 2.5% sucrose, and 3 mM NaCl in 0.1M sodium cacodylate buffer (pH 7.4), followed by postfixation with 2% osmium tetroxide in 0.1 M sodium cacodylate buffer (pH 7.4). Following fixation, cells were dehydrated in graded ethanol (from 70 to 100%) and embedded in EMBed-812 resin (Electron Microscopy Sciences, Hatfield, PA). Thin sections (90 nm) were cut with a diamond knife using a Reichert-Jung Ultracut E ultramicrotome (Leica, Wetzlar, Germany) and picked up with copper slot (1 × 2 mm) grids. Grids were stained with 2% uranyl acetate and 0.03% lead citrate and then viewed using a Zeiss Libra 120 transmission electron microscope with a Veleta camera (Olympus, Muenster, Germany).

### Enrichment of HIV-1.

To enrich for HIV-1, culture medium containing HIV-1 plus EVs were collected HIV-1-infected H9 cells, and followed by centrifugation at 3,000 *g* for 15 min, 10,000 *g* for 30 min, and 100,000 *g* for 3 h. Suspension of HIV-1 plus EVs mixture was applied to Dynabeads M-450 Epoxy (50 μL, Thermo Fischer Scientific) coupled with antibodies of anti-CD63 (5 μg, Santa Crus Biotechnology) and CD9 (5 μg, Santa Crus Biotechnology) and incubated overnight at 4 °C. HIV-1 fraction was collected from the bead by flow through, and the remaining bead-bound materials were used as EVs.

### Lipidomics.

A crude lipid fraction was extracted from HIV-1 particles using a modified Bligh and Dyer procedure. In brief, virion particles were gently mixed in a glass vial with ddH2O (to make up 1 mL suspension) and 2.9 mL methanol/dichloromethane (2:0.9, v/v) containing the following twelve internal standards: Cer d18:1/12:0 −6 ng/mL, SM d18:1/12:0 −0.3 ng/mL, GlcCer d18:1/12:0 −3.3 ng/mL, LacCer 18:1/12:0 −10.6 ng/mL, d5-DAG d16:0/16:0 −12.5 ng/mL, d5-TAG 16:0/18:0/16:0 −0.5 ng/mL, cholesteryl-d7 ester 16:0 −30 ng/mL, PA d12:0/12:0 −1025 ng/mL, PC 12:0/12:0 −0.2 ng/mL, PE d12:0/12:0 −1.6 ng/mL, PG d12:0/12:0 −200 ng/mL, and PS d12:0/12:0 −900 ng/mL. To obtain a biphasic mixture, an additional 1 mL of ddH2O and 900 μL dichloromethane was added and vortexed. The resultant mixture was incubated on ice for 30 min and centrifuged (10 min, 3000 *g*, 4 °C) to separate the organic and aqueous phases. The organic phase was removed and stored at −20 °C. Just prior to analysis, 1 mL of the organic layer was dried using a nitrogen evaporator (Organomation Associates, Inc., Berlin, MA, USA) and resuspended in 150 µl of running solvent (dichloromethane:methanol (1:1) containing 5 mM ammonium acetate), and 5 mg/mL of ceramide C17:0 used to track instrument performance. Lipid analysis was conducted in MS/MSALL mode on a TripleTOF 5600 (AB SCIEX, Redwood City, CA) time-of-flight mass spectrometer (TOF MS). Samples (50 μL injection volume) were direct infused by HPLC at a constant flow rate of 7 µL/min using an LC-20AD pump and SIL-20AC XR autosampler (Shimazu, Canby, OR). The mass spectrometer was operated at a mass resolution of 30,000 for TOF MS scan and 15,000 for product ion scan in the high sensitivity mode and automatically calibrated every 10-sample injections using APCI positive calibration solution delivered via a calibration delivery system (AB SCIEX). Source parameters were optimized and set as follows: ion source gases at 15 (GSI) and 20 psi (GS2), curtain gas at 30 psi, temperature at 150 °C, positive ion spray voltage at 5,500 V, declustering potential at 80 V, and precursor ion collision energy at 10 V. Each sample was run in duplicate in positive ion mode. An initial TOF MS scan provided an overview of the total lipid content at an accumulation time of 5 s. Precursor ions were selected by sequential 1-thomson mass steps from 200 to 1,200 m/z, and the analytes in each 1-thomson step were introduced into the collision chamber and fragments were produced by collision-induced dissociation and identified by TOF with a scan range of 100 to 1200 m/z (accumulation time of 450 ms). The collision energy for each MS/MS step was 40 V. The TOF MS and MS/MSALL data obtained were postaligned to internal standards using Analyst TF 1.8 (AB SCIEX) with mass error less than 5 ppm. The LipidView (version 1.3, AB SCIEX, Concord, Ontario, Canada) database was used for the identification and annotation of lipid species based on the precursor and fragment matchings from the experimental pooled sample runs. Lipid identifications were validated using a pooled (sample aliquots from all the experimental samples) sample extract and sequentially analyzed eight times. Each lipid species identified had to appear in seven out of the eight replicates and have a coefficient of variation below 20% in order for it to be included in the targeted lipid list. The resultant targeted lipid list was then used to identify these prevalidated lipid species among experimental samples using MultiQuant software (version 3.0, AB SCIEX, Concord, ON, Canada). Each sample was run in duplicate, and averaged values of each lipid were used for analysis. For statistical analysis purpose, missing lipid species in some experimental samples (less than 30% to the total number of samples) were replaced with the lowest intensity that was observed for the particular class of lipid in the corresponding group.

### Animals.

NOD.Cg-*Prkdc^scid^ Il2rg^tm1Wjl^*/SzJ mice (NSG, Jackson, Bar Harbor, ME) were housed in a temperature- and humidity-controlled room under a 12-h light cycle. All procedures were conducted in accordance with NIH guidelines for the Use of Animals and Humans in Neuroscience Research and approved by the Johns Hopkins Institutional Animal Care and Use Committee. For maintaining NSG colonies, females homozygous for both the Prkdc^scid^ and *Il2rg^tm1Wjl^* alleles were bred to males homozygous for Prkdc^scid^ and hemizygous for *Il2rg^tm1Wjl^*. Genetic mutation or knock out was validated using primer sets of forward primer 5′-TGTAACGGAAAAGAATTGGTATCCA-3′ with reporter 5′-ACATAAAATACGCT ATGCTAAG-3′ and reverse primer 5′-GTTGGCC​CCTGCTAACTTTCT-3′ with reporter 5′-ATAAA ATACGCTAAGCTAAG-3′ (Prkdc^scid^) and forward primer 5′-CCAAAGAGATTACTTCTGGCTGTC A-3′ with reporter 5′-TATCGATAAGC​TTGATATCTTC-3′ and reverse primer 5′-CCCCTACCCGG TAGAATTGAC-3′ (*Il2rg^tm1Wjl^*). Bone marrow–liver–thymus (BLT) mice in which human T cells can develop in the context of a human thymic microenvironment (69) were obtained from Humanized Mouse Core Facility, CCTI, CUMC, Columbia University.

### HIV-1 Replication in Humanized Mice.

At postnatal day 0, NSG mice (Jackson, Bar Harbor, ME) were exposed to γ-irradiation (10Gy, 137Ce source Gammacell 40, Nordion, Ottawa, ON, Canada), followed by intrahepatic injection of 2.0 × 10^5^ human umbilical cord blood-derived CD34+ hematopoietic stem cells (HSCs, Stem Cell Technology Vancouver, BC, Canada). Reconstitution of human immune cells in NSG-mice was verified 20 to 22 wk after transplantation by fluorescence-activated cell sorting (MACSQuant Analyzer 10; Miltenyl Biotec, Bergisch Gladbach Germany) using human-specific antibodies for hCD45-APC (1:50; Thermo Fisher Scientific), hCD3-BV650 (1:100; BioLegend), hCD14-PE/Cy7 (1:100; BioLegend), hCD19-BV421 (1:100; BioLegend), hCD4-PerCP (1:100; BioLegend), and hCD8-APC/Fire750 (1:100; BioLegend). Humanization was quantified as a ratio for each of these human cell types to mouse CD45-FITC (1:200; Thermo Fisher Scientific). Mice with less than 5% humanization were not included for experimentation. At 20 to 22 wk after humanization, mice were intraperitoneally infected with HIV-11-Ada (10,000 TCID_50_). Blood was collected weekly from the facial vein, and plasma viral load was calculated as follows: Viral (RNA) was isolated from plasma using the QuantiTect Virus Kit (Qiagen). Viral loads were determined by (qRT-PCR) using primers in the SIV *gag* region. The reaction included a nontemplate control and nonenzyme control, and samples were analyzed on an Applied Biosystem 7,300 Real-Time PCR system (Life Technology, Carlsbad, CA). Cycling parameters were 1 cycle at 50ºC for 30 min, 1 cycle at 95 °C for 15 min, and 45 cycles at 94 °C for 15 s, 55 °C for 30 s, and 60 °C for 30 s. The dissociation stage was 95 °C for 15 s, 60 °C for 1 min, and 95 °C for 15 s. CT values were converted to RNA copies/ml and compared with CT values from known copy number of standard HIV RNA (gifted from Janice Clements).

### NSMase2 Inhibitor Treatment.

HIV-1-infected humanized NSG mice were administered PDDC (10 mg/kg; reconstituted in 5% dimethylsulfoxide (DMSO), 5% Tween 80, and 90% saline intraperitoneal (IP) once daily beginning 5 wk following HIV-1 infection. This dose was chosen because PDDC was reconstituted in 5% dimethylsulfoxide (DMSO), 5% Tween 80, and 90% saline. Mice administered vehicle (5% DMSO and 5% Tween 80 in saline) or compound five (inactive structural analog of PDDC) were used as controls. Mice administered ARV cocktails containing zidovudine (azidothymidine, AZT, 45 mg/kg, Sigma), lamivudine (45 mg/kg, Sigma), and indinavir (45 mg/kg, Abcam, Cambridge, MA) were used for comparison against experimental drugs. Facial vein blood draws were conducted weekly for quantitation of human immune cell counts and plasma viral loads. Vorinostat (100 mg/Kg, Selleckchem, Houston, TX) and iBet151 (20 mg/kg, Selleckchem) were daily administered (IP) to reactivate latent reservoirs.

### NSMase2 Inhibitor Toxicity Assessments.

Gross alterations in autonomic or somatomotor systems of HIV-1-infected NSG mice treated with PDDC were subjectively assessed using a modified SmithKline Beecham, Harwell, Imperial College, Royal London Hospital, phenotype assessment (SHIRPA) test battery as previously described (70). Mice were placed individually into a new cage and visually examined for signs of autonomic nervous system dysfunction including ptosis, exophthalmus, miosis, mydriasis, corneal reflex loss, pinna reflex loss, piloerection, hyperventilation, writhing, tail erection, lacrimation, salivation, and vasodilation, as well as somatomotor disturbances including hyperlocomotion, convulsion, arching, tremor, spraddle, leg weakness, escape loss, placing loss, grasping loss, righting loss, catalepsy, and tail pinch reflex. Each behavior was evaluated using a rating scale from 0 to 2 where 0 = robust effect, 1 = modest effect, and 2 = no effect. Grip strength was assessed using an inverted hang protocol, where time to fall was measured for up to 60 s. Scores for each test were rated by two independent investigators blinded to the experimental treatment. Potential drug toxicities were assessed using the VetAce Clinical Chemistry system (Alfa Wasserman Diagnostic Technologies LLC., West Caldwell, NJ) by the Johns Hopkins School of Medicine Diagnostic & Phenotyping core. Blood was collected by cardiac puncture using heparin (Sigma)-coated syringe and EDTA tubes (BD) and centrifuged at 3,000 *g* for 25 min at 4 °C to obtain plasma. The clinical chemistry panel was focused on liver and kidney toxicity including enzyme activities of alkaline phosphatase, alanine aminotransferase, aspartate aminotransferase, and levels of albumin, glucose, creatine, blood urea nitrogen, and calcium.

### Adoptive Transfer.

HIV-1-infected humanized mice in which viral replication was suppressed by PDDC or ARVs for 10 weeks were killed and their splenocytes were harvested. Spleen isolated from each mouse was placed in a 70-µm cell strainer attached to a 50-mL tube and pressed through the strainer using the 3-mL syringe plunger. After washing cells through the strainer with HBSS, cells were isolated by centrifugation at 300 *g* for 10 min. Cells were lysed with prewarmed BD Pharm Lyse lysing solution (BD, San Jose, CA) at 37 °C for 5 min. After washing with HBSS, the cells were harvested by centrifuge at 300 *g* for 10 min, resuspended in HBSS, and transferred into uninfected humanized mice (2.0 × 10^6^/ mouse, I.P.). Blood from adoptively transferred humanized mice was weekly collected from the facial vein, and viral load was measured using RT-PCR as described above.

*RT-PCR.* Total RNAs from HIV-1-infected cells or tissues from HIV-1-infected humanized mice were extracted using the RNeasy Mini Kit according to the manufacturer’s instructions (Qiagen, Valencia, CA). RNA (1 µg) was reverse transcribed, and cDNA was amplified for Smpd1 using forward primer 5′-TGGCTCTATGAAGCGATGG-3′, reverse primer 5′-TGGGGAAAGAGCATAGAACC-3′; Smpd2 using forward primer 5′-CCAGTTCATCCACCACACAT-3′, reverse primer 5′-TCTTCTGGGTGCATGTTGAG-3′; Smpd3 using forward primer 5′-GCCGGCCCTACATCTATTC-3′, reverse primer 5′-CCCTTCCATTCACTGAGCA-3′; Smpd4 using forward primer 5′-GGCTGAGATGAT​TCAGAAAGGT-3′, reverse primer 5′-CCGTGAAGAGTCGGTGCT-3′; and actin using forward primer 5′-GCTACGAGCTGCCTGACG-3′, reverse primer 5′-GGCTGGAAGAGTGCCTCA-3′. Each 96-well plate included a nontemplate control, and each sample was analyzed in triplicate on an Applied Biosystem 7,300 Real-Time PCR system (Life Technology). PCR cycle parameters were as follows: 1 cycle at 50 °C for 2 min, 1 cycle at 95 °C for 10 min, and 40 cycles at 95 °C for 15 s and 60 °C for 1 min. The dissociation stage was 95 °C for 15 s, 60 °C for 1 min, and 95 °C for 15 s.

### Statistics.

All of the results were analyzed using one-way ANOVA followed by Tukey post hoc analyses when group differences were significant. Results are expressed as mean ± SD or mean ± SE as indicated.

## Supplementary Material

Appendix 01 (PDF)

## Data Availability

All study data are included in the article and/or *SI Appendix*.
