# Supplementary material for: Inhibition of neutral sphingomyelinase 2 impairs HIV-1 envelope formation and substantially delays or eliminates viral rebound
Source: Proc Natl Acad Sci U S A. 2023 Jul 5;120(28):e2219543120. doi: 10.1073/pnas.2219543120 (PMC10334757; doi:10.1073/pnas.2219543120)
Supplement: Supplementary file 1 — Appendix 01 (PDF) [file pnas.2219543120.sapp.pdf]

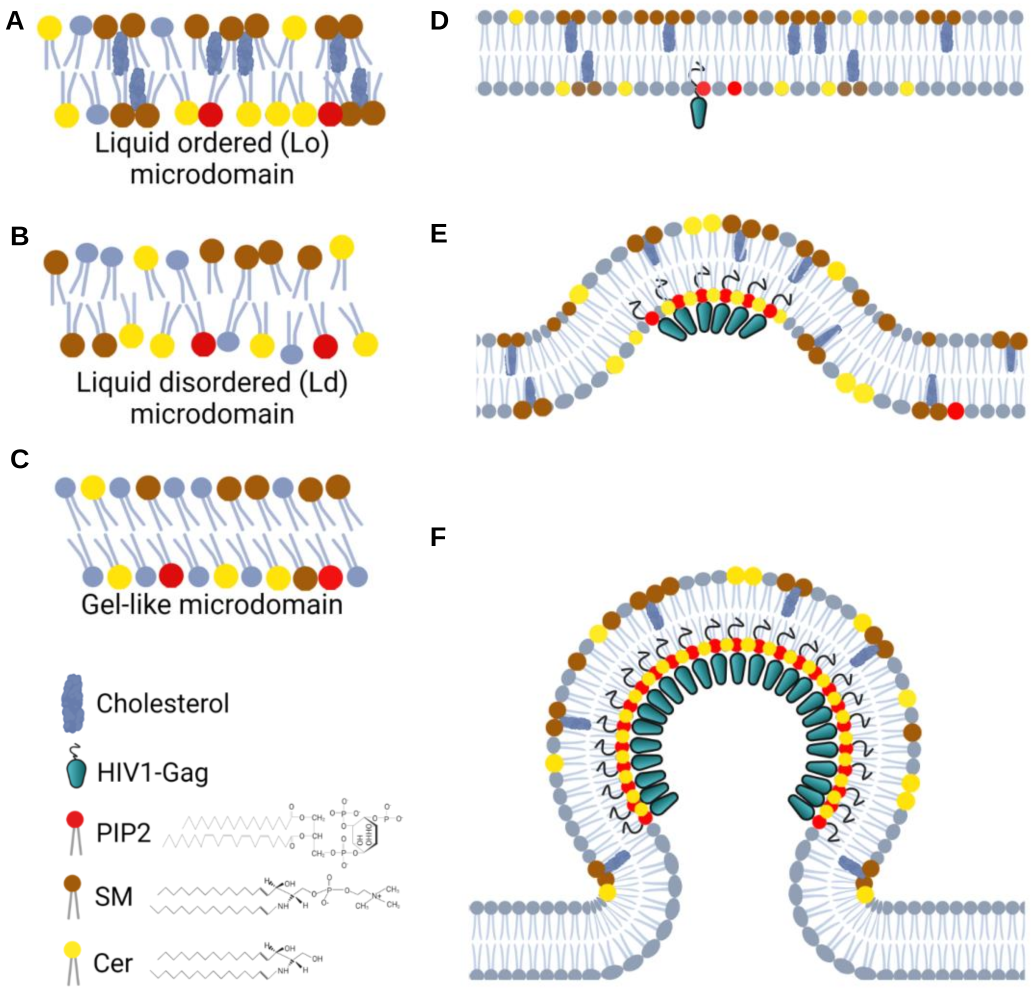


**Supplementary Figure 1. Biophysical properties of plasma membranes. (A)** Liquid ordered (Lo) domains are membrane microdomains that are enriched with saturated lipids such as sphingomyelin, saturated phospholipids, and cholesterol. The acyl chains of highly saturated lipids are relatively straight allowing for the lipids to pack closely together with limited lateral mobility in the membrane. Lo domains are stabilized by cholesterol that binds sphingomyelins together. **(B)** Liquid disordered (Ld) domains of the plasma membrane are more fluid and contain a higher content of unsaturated lipids. The acyl chains of unsaturated lipids have multiple double bonds that create kinks in the acyl chains that prohibit close packing. **(C)** Gel-like microdomains are created when the sphingomyelin of Lo domains is converted to cearmide. They are not as rigid as Lo domains and exhibit some flexibility. **(D)** HIV-1 Gag binds to $PiP_2$ on the inner leaflet of the plasma membrane and begins to form cholesterol and $PIP_2$ enriched domains **(E)** Gag multimerization and the selective recruitment of proteins that prefer Lo domains apply physical pressure to the membrane that begins to curve outwards **(F)** In order for the membrane to form a spherical shape, and wrap the assembling Gag lattice, the lipid composition of the inner and outer membranes are modified to include lipids that have chemical structures which facilitate membrane curvature (inset) such as ceramide that forms spontaneous negative curvature. Membrane curvature is regulated by lipid composition, selective recruitment of proteins with physical properties that facilitate curvature, protein motifs such as BAR domains, protein scaffolding, and cytoskeleton remodeling.

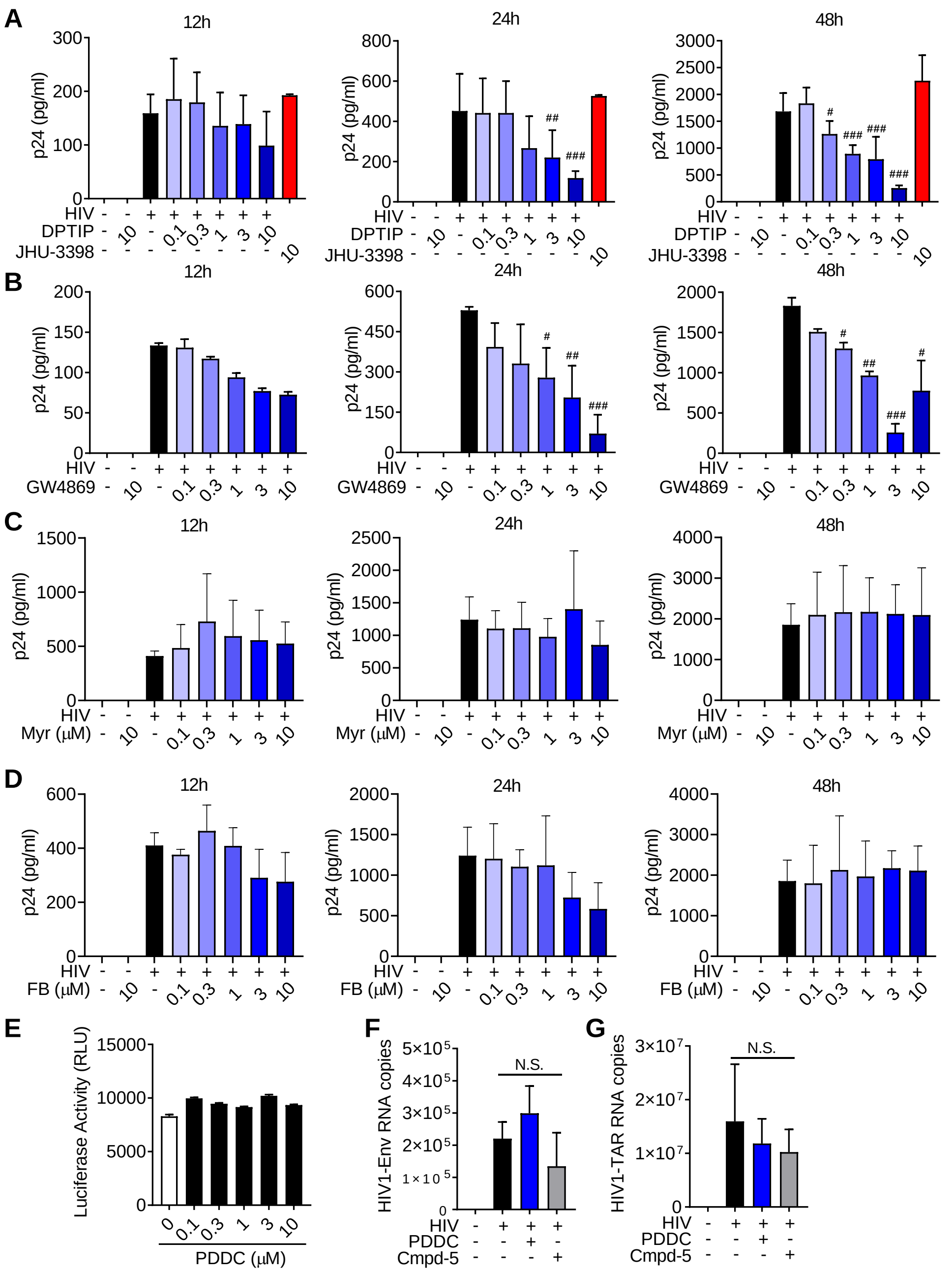

**Supplementary Figure 2. Inhibition of nSMase2 impairs HIV biogenesis but does not interfere with HIV entry or transcription.** Quantitation of p24 levels in media of $HIV_{RF}$ infected H9 cells treated with a doses escalation of the nSMase2 inhibitors (**A**) DPTIP (0-10 µM) and its inactive structural analog JHU-3398 (10 µM), (**B**) GW4869 (0-10 µM), (**C**) a ceramide synthase inhibitor, fumonisin B1 (0-10 µM), (**D**) a serine palmitoyl transferase inhibitor, myriocin (0-10 µM). HIV p24 was measured in culture media at 12-48h after inhibitor treatments. (**E**) Quantitative analysis of luciferase in $HIV_{Bal}$ infected TZM-bl cells expressing a luciferase gene under control of HIV-1 LTR following treatment with a dose escalation of PDDC (0-10 µM) . PCR analysis of (**F**) HIV1-Env and (**G**) HIV1-TAR in $HIV_{RF}$ infected H9 cells treated PDDC (3 µM) or Cmpd-5 (10 µM) for 6h. # = p < 0.05, ## = p < 0.01, ### = p < 0.001 compared to uninfected untreated cells. Data are mean ± S.D of n=3 per independent experiments/condition. One-Way ANOVA with Tukey's post-hoc analysis.

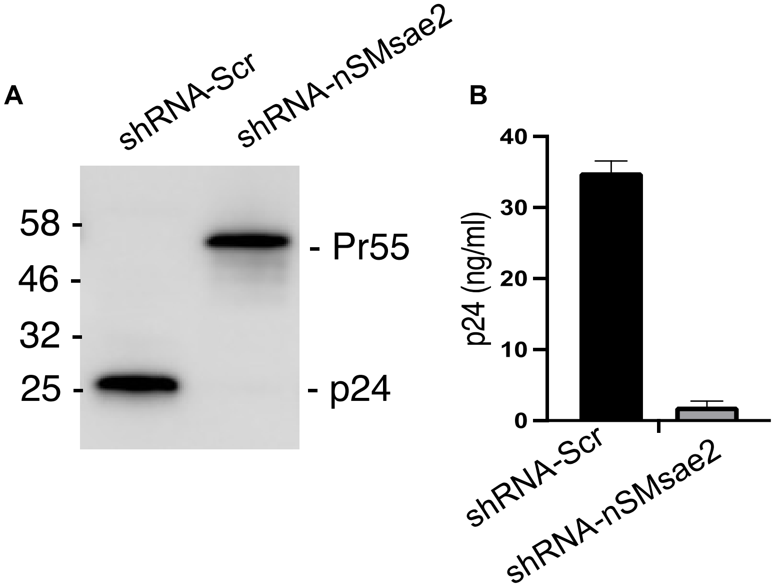


**Supplemental Figure 3. Molecular interference of nSMase2 is associated with a lack Gag processing. (A)** 293T cells were transduced with lentiviral particles carrying scrambled (Scr) or nSMase2 (nSM)-specific shRNA. One-day posttransduction, cells were transfected with pNL4-3 and 24 h posttransfection cell and virus lysates were prepared and subjected to western blot analysis with HIV-Ig. The mobility of molecular mass standards is shown on the left of each blot in kDa. Positions of the Gag precursor Pr55Gag (Pr55) and p24 are labeled on the right. A representative image from two independent experiments is shown. **(B)** p24 levels were determined as in Fig. 2. Data shown are ± SD from two independent experiments.

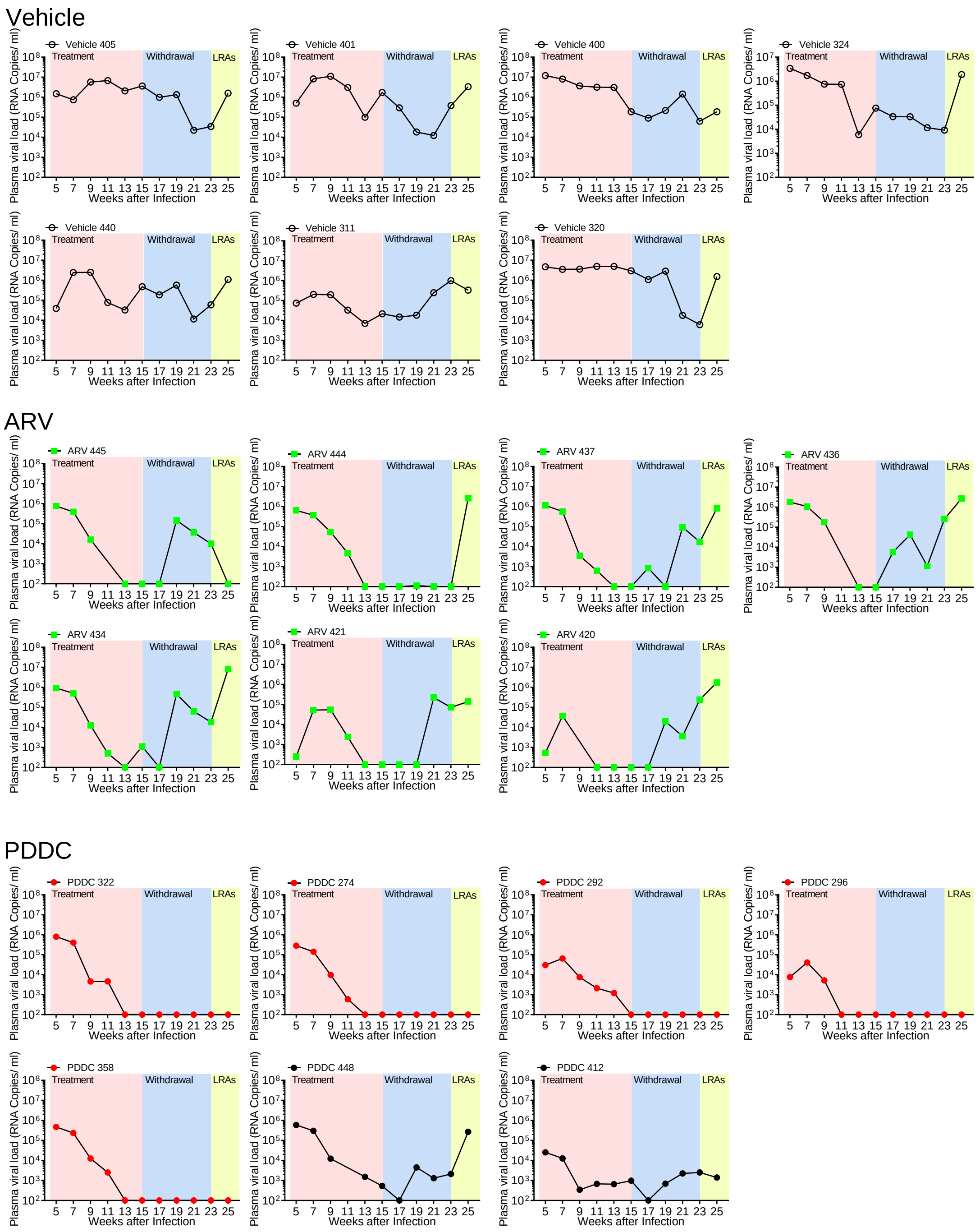


**Supplementary Figure 4. Weekly plasma p24 levels for individual HIV infected huNSG mice.** HuNSG mice were infected with $HIV_{ADA}$ and treated with vehicle (open black circles, n=7), antiretrovirals (ARV; azidothymidine, lamivudine, and indinavir, I.P., 45 mg/kg/day, closed green circle, n=7), PDDC (I.P.,10 mg/kg, closed red circles, n=7) followed by 8 weeks of withdrawal and 2 weeks of latency reversal (LRAs) with vorinostat (100 mg/kg, I.P.) and a Bromodomain inhibitor (iBet, 20 mg/kg, I.P.). Five out of seven mice treated with PDDC achieved plasma HIV loads below detection limits and did not exhibit viral rebound following termination of drug treatment (red closed circles). Two out of seven PDDC treated mice did not achieve plasma HIV loads below detection limits and showed viral rebound during withdrawal (black closed circles).

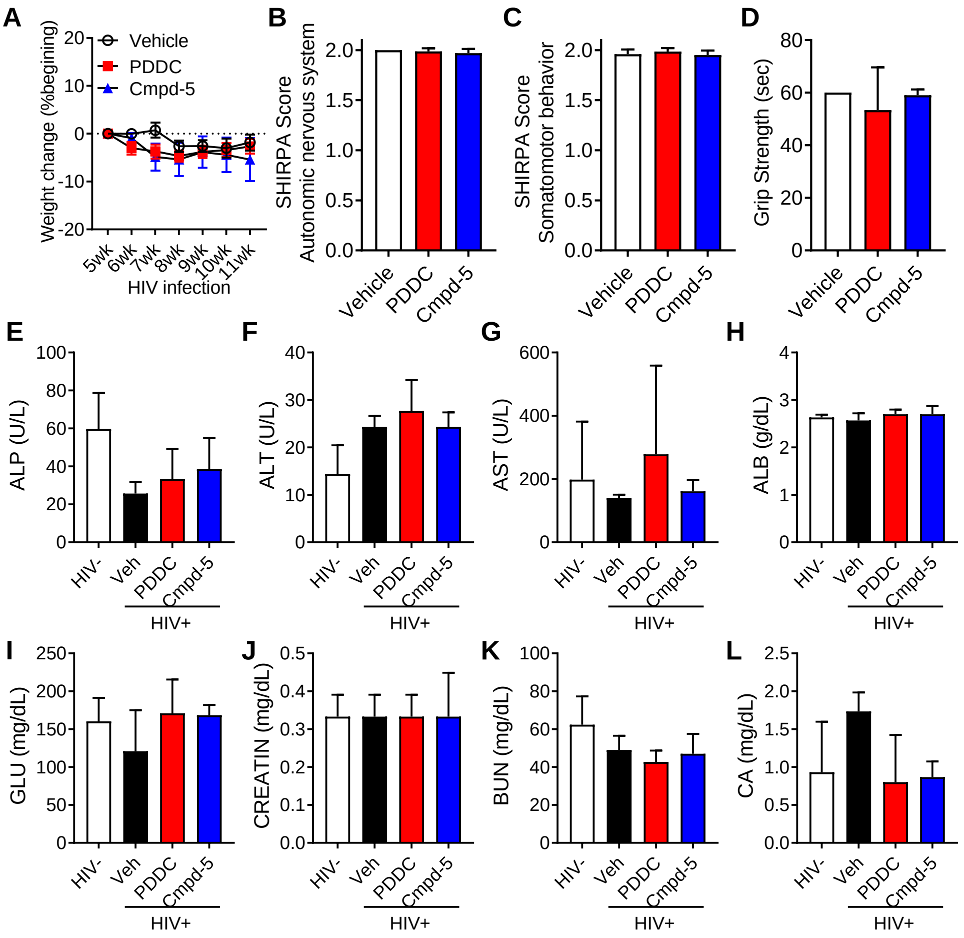


**Supplementary Figure 5. PDDC treatment does not alter body weight, behavior or clinical chemistry.** (**A**) Weight of HIV infected humanized NSG mice administered vehicle, PDDC or Cmpd-5 were measured weekly and presented as percent change based on weight before treatments. (**B**) Quantitation autonomic nervous system including ptosis, exophthalmus, miosis, mydriasis, corneal reflex loss, pinna reflex loss, piloerection, hyperventilation, writhing, tail erection, lacrimation, salivation, and vasodilation, (**C**) somatomotor disturbances including hyperlocomotion, convulsion, arching, tremor, spraddle, leg weakness, escape loss, placing loss, grasping loss, righting loss, catalepsy, and tail pinch reflex, and (**D**) grip strength in mice administered vehicle, PDDC, or Cmpd-5 daily for 6 weeks. Each category was evaluated using a rating scale from 0 to 2 with 0=robust effect, 1=modest effects, 2=no effect. Quantitation of enzyme activities or levels of the indicated metabolites. (**E**) Alkaline phosphatase (ALP), (**F**) Alanine aminotransferase (ALT), (**G**) Aspartate aminotransferase (AST), (**H**) Albumin (ALB), (**I**) Glucose (GLU), (**J**) Creatin, (**K**) Blood urea nitrogen (BUN), (**L**) Calcium (CA). Data are mean ± S.D (n=4-6).

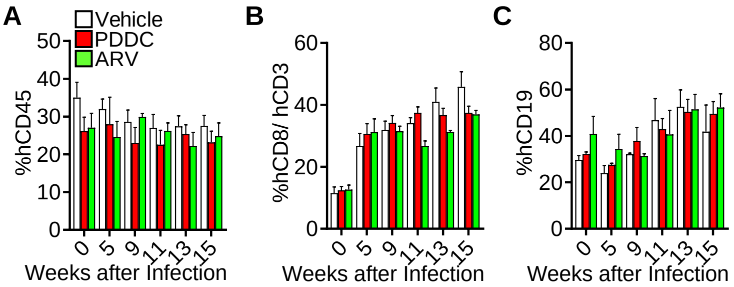


**Supplementary Figure 6.** huNSG mice infected with $HIV_{ADA}$ and treated with Vehicle, PDDC (I.P.,10 mg/kg, n=7), antiretroviral therapy (ART; azidothymidine, lamivudine, and indinavir, I.P., 45 mg/kg/day, n=7), or vehicle (n=7) for 10 weeks followed by 8 weeks of withdrawal and 2 weeks of latency reversal (LRAs) with vorinostat (100 mg/kg, I.P.) and a bromodomain inhibitor (iBet, 20 mg/kg, I.P.). Quantitation of human (**A**) CD45+, (**B**) CD8/CD3+, and (**C**) CD19+ cells for the indicated treatment conditions and time points in $HIV_{ADA}$ infected huNSG mice.

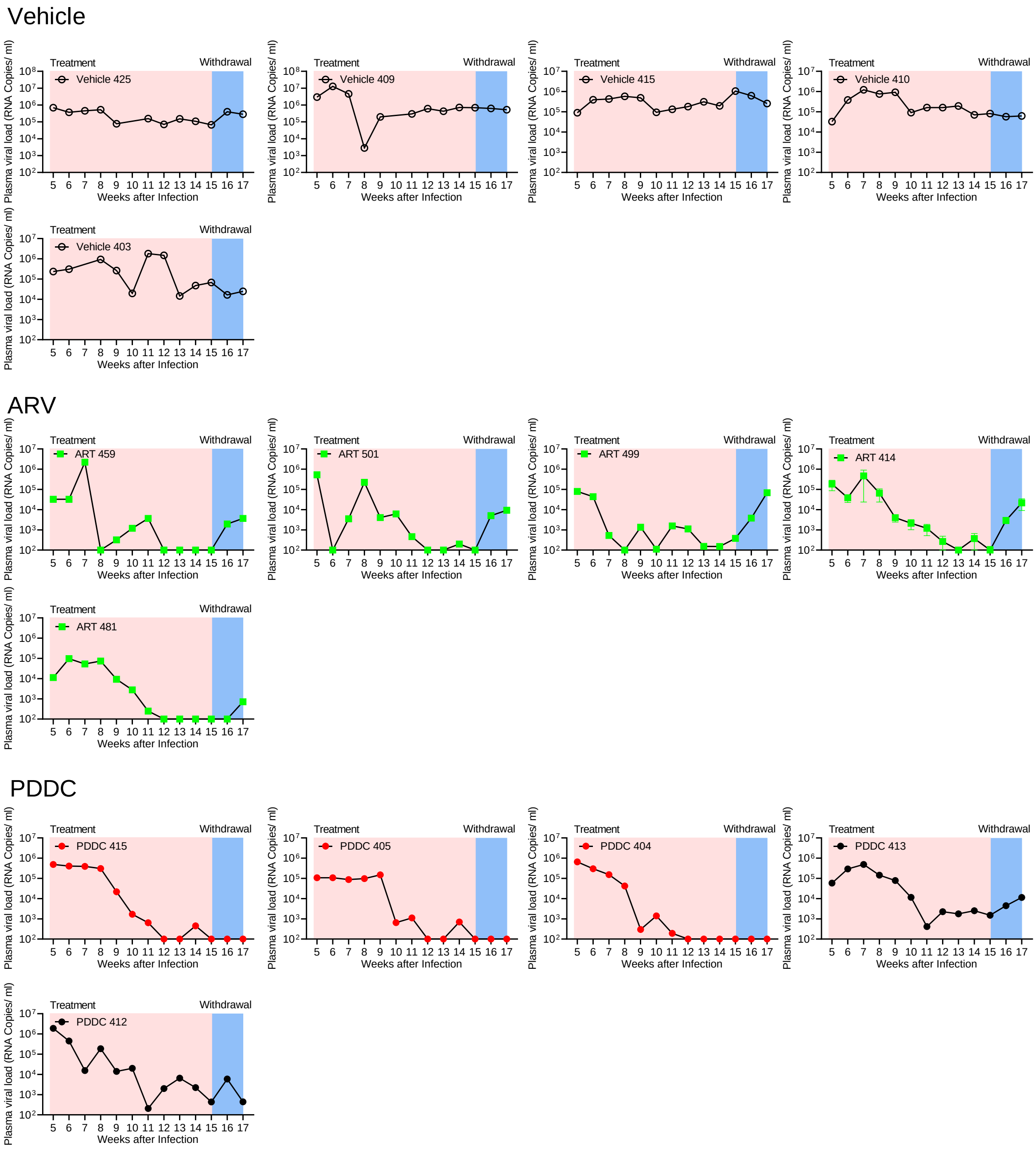


**Supplementary Figure 7. Weekly plasma HIV loads for individual HIV infected BLT mice.** BLT mice were infected with $HIV_{ADA}$ and fed chow containing PDDC (500 mg/Kg), or combination of antiretrovirals (Tenofovir disoproxil fumarate, 1,560 mg/Kg; Emtricitabine, 1,500 mg/Kg; Rategravir, 600 mg/Kg) for 10 weeks, followed by return to normal chow for 2 weeks. All mice treated with ARVs achieved plasma HIV loads below detectable limits and all mice showed viral rebound following return to normal chow. Three out of five mice treated with PDDC achieved plasma HIV loads below detectable limits and did not exhibit viral rebound when returned to normal chow (red closed circles). Two out of the five mice treated with PDDC did not achieve plasma HIV levels below detectable limits during inhibitor treatment and exhibited viral rebound after return to normal chow (black closed circles).

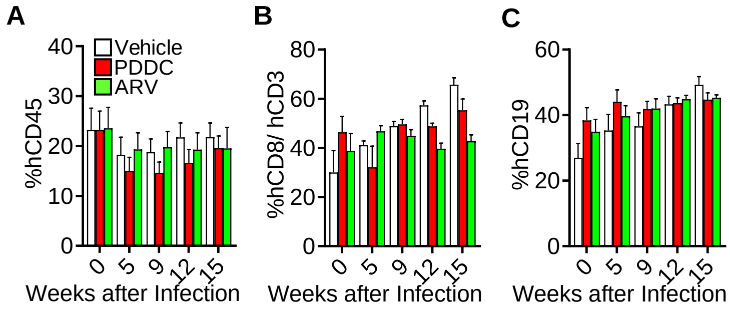


**Supplementary Figure 8.** BLT mice were infected with $HIV_{ADA}$ and treated with PDDC (I.P.,10 mg/kg, n=7), antiretroviral therapy (ART; azidothymidine, lamivudine, and indinavir, I.P., 45 mg/kg/day, n=7), or vehicle (n=7) for 10 weeks followed by 8 weeks of drug withdrawal. Quantitation of human (**A**) CD45+, (**B**) CD8/CD3+, and (**C**) CD19+ cells for the indicated treatment conditions and time points.

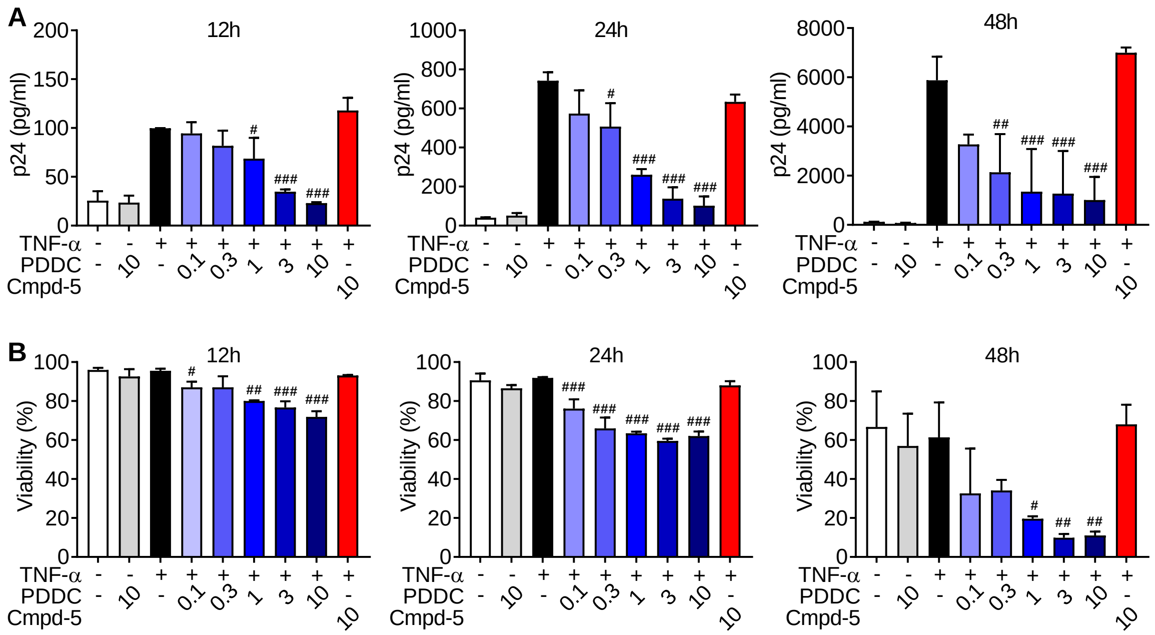


**Supplementary Figure 9. Inhibition of nSMase2 produced a selective death only in HIV infected cells with actively replicating HIV.** (**A**) Quantitation of p24 levels in culture media and (**B**) viability of U1 cells. Low level basal production of HIV and viability of constitutively HIV infected U1 cells was not reduced by treatment with PDDC for 24h. In U1 cells where HIV replication was induced by treatment with TNF-α (500 ng/ml) a dose escalation of PDDC (0.1-10μM) produced a dose related reduction in p24 levels and viability over time of treatment (12, 24, 48h). These data suggest that active HIV replication is required for PDDC mediated cytotoxicity. Data are mean ± S.D of n=3 independent experiments per condition. # = $p < 0.05$, ## = $p < 0.01$, and ### = $p < 0.001$ compared to TNF-α only group. One-Way ANOVA with Tukey's post-hoc comparisons.
